# Forecasting monthly AIDS incidence in China via LSTM-CNN parallel fusion: a comparative study of 10 predictive models

**DOI:** 10.3389/fpubh.2026.1873637

**Published:** 2026-07-29

**Authors:** Chengcheng Li, Jinfeng Li, Shifeng Pang, Chao Rong, Ximan Ye, Xiaojie Lao, Maowei Chen

**Affiliations:** 1 Humanities and Management School, Zhejiang Chinese Medical University, Hangzhou, Zhejiang, China; 2 Department of Infectious Diseases, Wuming Hospital Affiliated to Guangxi Medical University, Nanning, Guangxi, China; 3 Department of Cardiology, Minzu Hospital of Guangxi Zhuang Autonomous Region, Nanning, Guangxi, China; 4 Department of Cardiology, Sir Run Run Shaw Hospital, Zhejiang University School of Medicine, Hangzhou, Zhejiang, China

**Keywords:** acquired immunodeficiency syndrome (AIDS), China, deep learning, hybrid models, incidence prediction, time series forecasting

## Abstract

**Background:**

Acquired immunodeficiency syndrome (AIDS) poses a significant global public health threat and ranks among the most fatal infectious diseases in China. Effective prevention hinges upon early surveillance and predictive warning systems. However, effective predictive tools specifically tailored to AIDS incidence in China remain scarce. This study aimed to systematically compare the applicability of multiple predictive models for forecasting monthly AIDS incidence in China, identify the relatively better-performing model within this dataset, and provide preliminary methodological references for AIDS surveillance.

**Methods:**

We collected monthly AIDS incidence data from China spanning January 2001 to September 2025. We developed and validated 10 predictive models across four categories: (I) traditional statistical approaches [seasonal autoregressive integrated moving average (SARIMA)]; (II) traditional machine learning algorithms [support vector regression (SVR) and Random Forest]; (III) deep learning models [gated recurrent unit (GRU), convolutional neural network (CNN), and long short-term memory (LSTM)]; and (IV) hybrid deep learning fusion models [GRU-LSTM serial fusion, LSTM-CNN parallel fusion, LSTM-GRU-Attention, and LSTM-CNN-Attention]. Data were partitioned into training and validation sets using a 7:3 split ratio. Model performance was assessed using the coefficient of determination (*R*^2^), mean absolute error (MAE), and mean absolute percentage error (MAPE).

**Results:**

Monthly AIDS incidence in China displayed marked seasonal patterns, peaking during winter months. All models captured the general temporal trend. The LSTM-CNN parallel fusion model demonstrated relatively superior generalization performance on the validation set. Error distribution analysis further confirmed that this model achieved optimal concentration, stability, and precision in its predictions. Projections from this optimal model indicate that AIDS incidence in China will follow an upward trajectory from 2026 to 2030, followed by a deceleration in growth rate; however, incidence will remain elevated.

**Conclusions:**

Among the 10 algorithms evaluated, the LSTM-CNN parallel fusion model exhibited relatively superior validation performance for forecasting monthly AIDS incidence in China, suggesting that hybrid deep learning architectures may offer certain advantages in capturing nonlinear dynamic characteristics within this specific dataset. Projections from this model indicate a slowly rising trend with fluctuations over the next decade.

## 1 Introduction

Acquired immunodeficiency syndrome (AIDS) is an infectious disease caused by infection with the human immunodeficiency virus (HIV). It typically leads to excessive activation and functional exhaustion of CD4+ T cells, and is characterized by CD4+ T-lymphocyte depletion, posing a severe threat to human health (1–3). Since its first report in the 1980s, AIDS has become a serious global public health and social problem (4). According to the latest data from the Joint United Nations Programme on HIV/AIDS (UNAIDS), as of the end of 2024, approximately 40.8 million people were living with HIV worldwide, with an estimated 1.3 million new infections and approximately 630,000 deaths due to AIDS-related diseases annually (5). In China, the situation regarding AIDS prevention and control is equally severe (6). Data from the National Center for AIDS/STD Control and Prevention, Chinese Center for Disease Control and Prevention show that as of December 31, 2024, China had 1.355 million existing HIV/AIDS patients, and 101,000 new HIV infections/AIDS cases were reported in 2024 (7). Despite substantial progress in global AIDS control, the overall situation remains severe, and many patients still lack access to treatment. AIDS poses a severe threat to human life, health, and public health, and the Chinese government has introduced multiple prevention and control measures. In 1995, China established the National Notifiable Disease Reporting System (NNDRS), and since 2004, this system has been used to monitor AIDS incidence (8).

Infectious disease forecasting involves the interaction of multiple dynamic factors, including pathogen variation, climatic factors, population mobility, and health policies, making it difficult for traditional passive surveillance systems to predict actual situations. Currently, the AIDS epidemic in China maintains a persistently high incidence, with sexual transmission having become the primary route, placing traditional surveillance systems under pressure to transform. Moreover, the long latency period of HIV and the large number of asymptomatic carriers make accurate estimation of incidence and reliable prediction particularly difficult (9). Furthermore, traditional disease surveillance systems suffer from weak infrastructure, rely on complex network architectures, and incur high management costs (10), and the COVID-19 pandemic has further exposed the shortcomings of real-time epidemic surveillance, highlighting the urgent need to develop robust infectious disease forecasting methods (11, 12).

A fundamental strategy for AIDS surveillance, prevention, and control involves early detection of abnormal epidemic trends and growth patterns, as well as timely identification and treatment of affected individuals (13). Early initiation of antiretroviral therapy (ART) in AIDS patients is an important means of improving treatment outcomes (14, 15). By analyzing trends in HIV infection and transmission, we can develop more accurate AIDS predictive models, which facilitate early identification of HIV-infected individuals and provide decision-making support for infectious disease prevention and control. Therefore, modeling and forecasting monthly incidence plays an important role in AIDS prevention and control. Currently, commonly used infectious disease surveillance and early warning models include gray prediction models (16), dynamic transmission models (17), mathematical models (18, 19), deep learning predictive models (20), and time-series forecasting models (21). In current research, both conventional modeling methods and deep learning approaches have achieved some success in predicting AIDS incidence. Some studies have explored the use of typical autoregressive integrated moving average (ARIMA) linear models to forecast AIDS morbidity and mortality in China (22). Additionally, both nonlinear back-propagation artificial neural network (BP-ANN) models and typical ARIMA models can be used to predict monthly AIDS incidence, with BP-ANN models outperforming conventional linear ARIMA models (23). Studies have reported that LSTM models typically achieve higher predictive accuracy than traditional models such as ARIMA and generalized regression neural networks (GRNN) for forecasting monthly AIDS incidence in Guangxi, China (20). To capitalize on the strengths of each model, some researchers have chosen to combine multiple modeling approaches. Empirical mode decomposition (EMD)–back-propagation neural network (BPNN) combined models can predict monthly AIDS incidence in Dalian, China (24). ARIMA-LSTM hybrid models can effectively predict pediatric AIDS incidence in East Asia (21). SARIMA-Prophet integrated models can predict AIDS incidence in Henan Province, China (25). However, these models have limitations, including limited data coverage, insufficient comparable models, and an inability to investigate multivariate data with diverse characteristics. They generally suffer from low predictive accuracy and poor generalizability.

To improve predictive accuracy, this study is based on monthly AIDS incidence data from China spanning January 2001 to September 2025 (a total of 297 months). We systematically compared the predictive performance of 10 models: SARIMA, SVR, RF, GRU, LSTM, CNN, and four fusion architectures. This study is characterized by a relatively long data time span and a comparatively comprehensive methodological framework, enabling preliminary evaluation of the applicability of different modeling strategies within this specific dataset, thereby identifying the relatively better-performing predictive model for monthly AIDS incidence in China and providing exploratory methodological references for AIDS surveillance.

## 2 Methods

**Figure 1:**
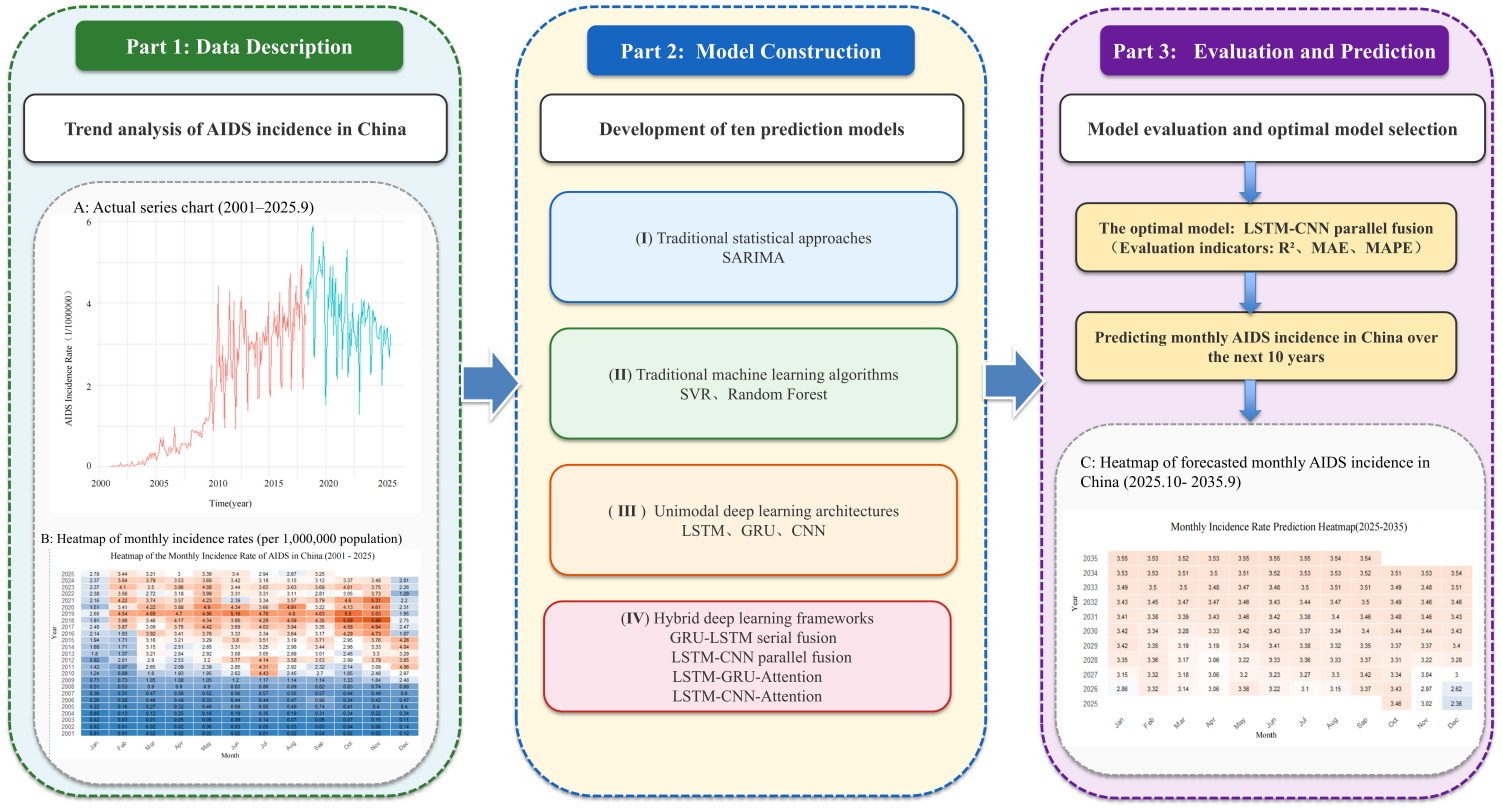
Study framework diagram. **(A)** Temporal sequence plot of actual incidence, visually presenting the overall trend and fluctuation characteristics of monthly AIDS incidence from January 2001 to September 2025; **(B)** Monthly incidence heatmap, displaying the spatiotemporal distribution pattern of incidence across months from 2001 to 2025 in matrix form; **(C)** Forecasting heatmap of projected monthly AIDS incidence in China from October 2025 to September 2035 based on the relatively better-performing LSTM-CNN model identified in this study.

This study follows a three-part technical framework comprising data description, model construction, and evaluation and forecasting (Figure 1). The first part involves trend analysis of monthly AIDS incidence data from China spanning January 2001 to September 2025. The second part entails model construction based on four categories of 10 predictive algorithms, with comparative evaluation of goodness-of-fit and predictive performance on the training and validation sets to identify the relatively better-performing model within this dataset. The third part employs the LSTM-CNN parallel fusion model to project monthly AIDS incidence in China over the next decade, with results serving as preliminary quantitative references for AIDS surveillance in China.

### 2.1 Data source

This study utilized monthly data on AIDS incidence in China spanning January 2001 to September 2025. These data were obtained from the official, publicly accessible AIDS database of the China Center for Disease Control and Prevention (https://www.chinacdc.cn/jksj/jksj01/) (26). Monthly incidence rates were calculated as follows: Monthly incidence rate = (Number of new AIDS cases in the month / Population at risk in the month) × 1,000,000. The average monthly incidence rate for the *i*-th month (*i* = 1, 2, ……, 12) was computed using the formula (1). Where: ${\bar{I}}_{i}$ represents the average monthly incidence rate for the *i*-th month (expressed per 1,000,000 population); *n*_*i*_ represents the number of sample years for the *i*-th month (sample size). For *i*∈[1, 9] (January–September): *n*_*i*_ = 25 (data from 2001–2025); for *i*∈[10, 12] (October–December): *n*_*i*_ = 24 (data from 2001–2024, as data for October–December 2025 were unavailable); *I*_*i, k*_ represents the monthly incidence rate for the *i*-th month in the *k*-th year (expressed per 1,000,000 population).


$$\begin{array}{l} {\bar{I}}_{i}=\;\frac{1}{n_{i}}\overset{n_{i}}{\underset{k=1}{\sum }}I_{i,k} \end{array}$$ (1)

### 2.2 Model construction

Time series forecasting modeling represents an important intersection of statistics and machine learning, with its core objective being the utilization of historical observational data to predict future trends and patterns. This study employed 10 models for fitting and prediction: traditional statistical models (SARIMA), traditional machine learning models (SVR and Random Forest), deep learning base models (GRU, CNN, and LSTM), and deep learning fusion models (GRU-LSTM serial fusion, LSTM-CNN parallel fusion, LSTM-GRU-Attention, and LSTM-CNN-Attention). The mathematical formulas, network architectures, and hyperparameter search spaces for each model are detailed in Supplementary Material Attachment 1 (S1–S10).

### 2.3 Model evaluation and validation protocol

**Figure 2:**
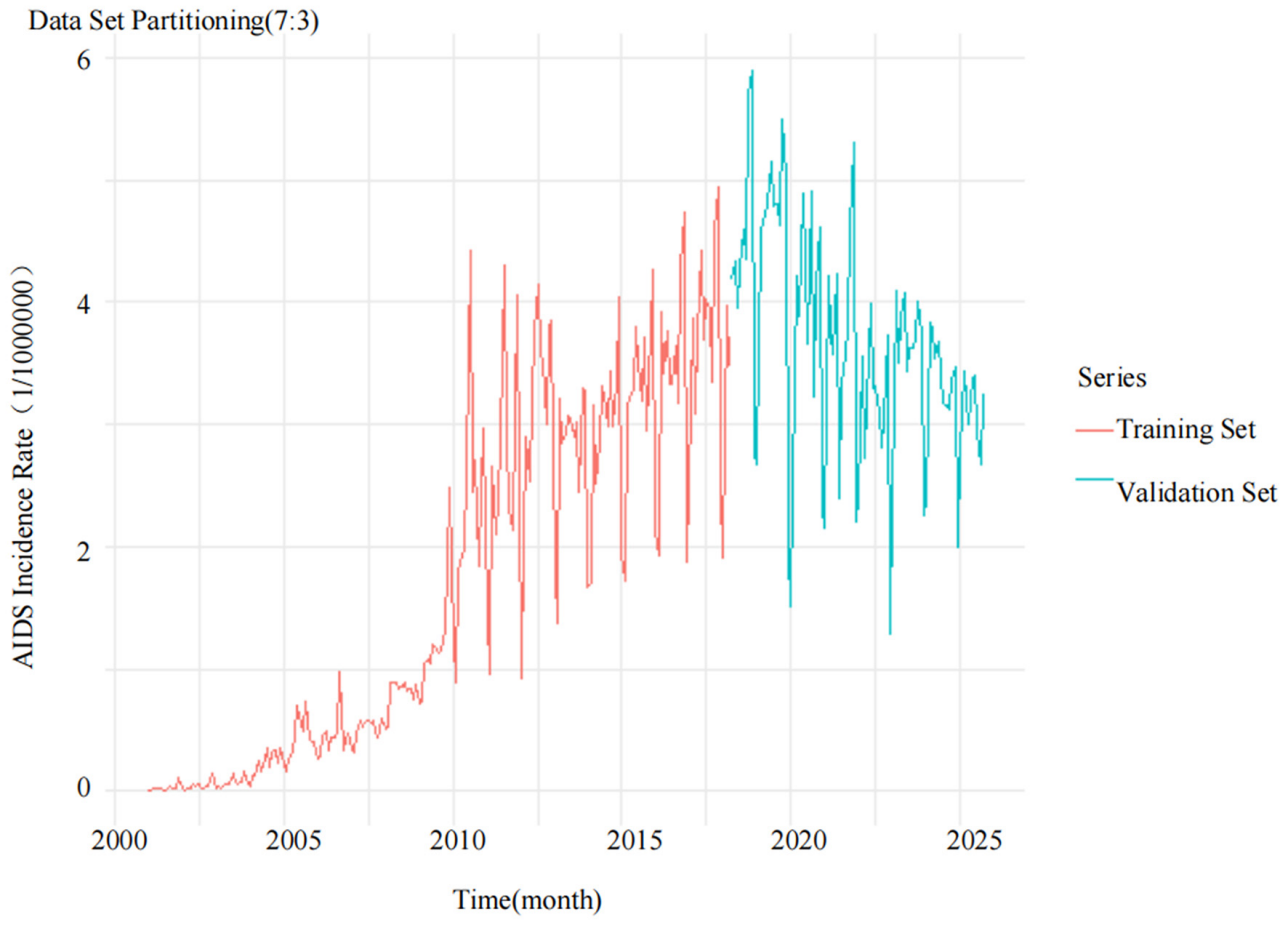
Temporal division of monthly AIDS incidence data in China using a 7:3 split ratio. The red curve denotes the training set (January 2001–June 2018), and the blue curve indicates the validation set (July 2018–September 2025). Y-axis: incidence rate per 1,000,000 population.

The dataset was partitioned into training and validation sets using a 7:3 ratio (Figure 2). Data from January 2001 to June 2018 (210 months) were allocated for model training, whereas data from July 2018 to September 2025 (87 months) served for external validation. Monthly incidence rates from October 2025 to September 2035 were projected using the best-performing model identified in this study. Predictive performance was assessed using the coefficient of determination (R^2^), mean absolute error (MAE), and mean absolute percentage error (MAPE) (27–30). Higher R^2^ values and lower MAE and MAPE values indicate superior predictive accuracy.

### 2.4 Data analysis

Core variables included monthly incident AIDS case counts, temporal indices (January 2001–September 2025), and incidence rates derived from corresponding population denominators. Outlier detection in the monthly incidence time series was performed using the Seasonal Hybrid Extreme Studentized Deviate (S-H-ESD) algorithm. Raw data underwent logical verification and consistency validation before importation into the analysis platform. Data preprocessing employed Min-Max normalization to scale data to the [0, 1] interval. Time-series samples were reconstructed using a sliding window approach, with the preceding 12 months (*n* = 12) of monthly incidence rates serving as input features to predict the incidence rate of the 13th month. The input tensor dimensions were [sample size, time steps, features], i.e., [sample size, 12, 1], and the output tensor dimensions were [sample size, 1].

Hyperparameter optimization for all models was performed exclusively on the training set data: conventional models employed grid search for systematic optimization within predefined parameter ranges, while deep learning models further partitioned the training set into a model training subset (80%) and a tuning validation subset (20%) for early stopping monitoring and hyperparameter selection. The final validation set was used for performance evaluation only after all hyperparameters were determined, with no involvement in any tuning process, ensuring no information leakage. To exclude chance results from a single partition and verify model robustness, this study additionally employed Expanding Window Walk-Forward Validation for supplementary validation. After each round of validation, the validation period data were incorporated into the training set to expand the window forward. R^2^, MAE, and MAPE were independently computed for each round, and data normalization parameters for each round were executed according to the aforementioned independent training set calculation workflow, ensuring no information leakage.

Model construction, training, and statistical analysis were implemented in MATLAB (R2023b, MathWorks, Natick, MA, USA) and R (4.5.0). Hyperparameter optimization employed grid search techniques: an initial coarse grid search was conducted across the predefined parameter space for each model, followed by fine-grained searching in promising regions, balancing parameter space coverage with model architecture complexity. The mathematical formulas, network architectures, and parameter search spaces for each model are detailed in Table 1.

**Table 1:** Overview of models evaluated in this study.

Type	Algorithm	Model function/architecture	Parameter search space
Classical statistics models	SARIMA	$\Phi_{P}\left(B^{s}\right)\phi_{p}\left(B\right)\nabla^{d}\nabla_{s}^{D}X_{t}=\Theta_{Q}\left(B^{s}\right)\theta_{q}\left(B\right)\varepsilon_{t}$	Seasonal period *s* = 12 Non-seasonal orders *p*[0~5], *d*[0~2], *q*[0~5] Seasonal order *P*[0~2], *D*[0~1], *Q*[0~2] Determined via AIC criterion
Traditional machine learning models	SVR	*f*(*x*) = ω^*T*^ϕ(*x*)+*b*	Kernel function = 'rbf' Gamma [0.001~1.0] C [0.1~100] Epsilon [0.01~1.0]
	Random forest	$ŷ=\frac{1}{\text{B}}\sum_{\text{b}=1}^{\text{B}}{\text{T}}_{\text{b}}\left(\text{X}\right)$	Number of trees [100~500] Max depth [5~30] Min samples split [2~10] Min samples leaf [1~5]
Deep learning models	GRU	Update gate: *z*_*t*_ = σ(*W*_*z*_·[*h*_*t*−1_, *x*_*t*_]+*b*_*z*_) Reset gate:*r*_*t*_ = σ(*W*_*r*_·[*h*_*t*−1_, *x*_*t*_]+*b*_*r*_) Candidate hidden state: ${\tilde{h}}_{t}=\tanh\left(W_{h}\cdot \left[r_{t}⊙h_{t-1},x_{t}\right]+b_{h}\right)$ $\text{Hidden state}:\;h_{t}=\left(1-z_{t}\right)⊙h_{t-1}+z_{t}⊙{\tilde{h}}_{t}$	Epochs [100~500] Batch size [1~64] Optimizer = [Adam] Learing rate [0.0001~0.01] Units per layer [16, 32, 64, 128] Dropout rate [0.1~0.5]
	LSTM	Forget gate: *f*_*t*_ = σ(*W*_*f*_·[*h*_*t*−1_, *x*_*t*_]+*b*_*f*_) Input gate: *i*_*t*_ = σ(*W*_*i*_·[*h*_*t*−1_, *x*_*t*_]+*b*_*i*_) $\text{Candidate cell state}:\;{\tilde{C}}_{t}=\tanh\left(W_{C}\cdot \left[h_{t-1},x_{t}\right]+b_{C}\right)$ $\text{Cell state}:\;C_{t}=f_{t}⊙C_{t-1}+i_{t}⊙{\tilde{C}}_{t}$ Hidden state: *h*_*t*_ = *o*_*t*_⊙tanh(*C*_*t*_)	Epochs [100~500] Batch size [1~64] Optimizer = [Adam] Learing rate [0.0001~0.01] Units per layer [16, 32, 64, 128, 256] Dropout rate [0.1~0.5]
	CNN	1 *Dconvolutional operation*: $\left(f*g\right)\left(t\right)=\sum_{\tau =1}^{k}f\left(\tau \right)g\left(t-\tau \right)$ Max pooling operation: $y_{i,j}=\underset{\left(m,n\right)\in R_{i,j}}{\max}x_{m,n}$	Epochs [100~500] Batch size [1~64] Optimizer = [Adam] Learing rate [0.0001~0.01] Units per layer [16, 32, 64, 128] Dropout rate [0.1~0.5]
Hybrid deep learning fusion models	GRU-LSTM serial fusion	GRU output as LSTM input: $h_{t}^{\left(G\right)}=GRU\left(x_{t},h_{t-1}^{\left(G\right)}\right)$ LSTM final hidden state: $\hat{y}=W_{o}\cdot h_{T}^{\left(L\right)}+b_{o}$	Epochs [100~500] Batch size [1~64] Optimizer = [Adam] Learing rate [0.0001~0.01] GRU hidden units [32, 64, 128] LSTM hidden units [32, 64, 128] Dropout rate [0.1~0.5]
	LSTM-CNN parallel fusion	CNN local features: *F*_*CNN*_ = *CNN*(*X*) LSTM temporal features: *F*_*LSTM*_ = *LSTM*(*X*) Fused features: *F*_*fuse*_ = *Concat*(*F*_*CNN*_, *F*_*LSTM*_) Prediction output: $\hat{y}=W_{f}\cdot F_{fuse}+b_{f}$	Epochs [100~500] Batch size [1~64] Optimizer = [Adam] Learing rate [0.0001~0.01] LSTM hidden units [32, 64, 128] CNN hidden units [16, 32, 64] Dropout rate [0.1~0.5]
	LSTM+GRU+attention	Attention weight calculation: $e_{t}=v^{T}\tanh\left(W_{h}h_{t}+b_{a}\right),\alpha_{t}=\frac{\exp\left(e_{t}\right)}{\overset{T}{\underset{j=1}{\sum }}\exp\left(e_{j}\right)}$ *Context vector*: $c=\overset{T}{\underset{t=1}{\sum }}\alpha_{t}h_{t}$ Prediction output: $\hat{y}=W_{o}\cdot c+b_{o}$	Epochs [100~500] Batch size [1~64] Optimizer = [Adam] Learing rate [0.0001~0.01] LSTM hidden units [32, 64, 128] GRU hidden units [32, 64, 128] Attention heads [1, 2, 4] Dropout rate [0.1~0.5]
	LSTM+CNN+attention	Spatiotemporal attention weights: $e_{t,f}=v^{T}\tanh\left(W_{f}\left[F_{CNN};F_{LSTM}\right]+b_{s}\right),\alpha_{t,f}=\frac{\exp\left(e_{t,f}\right)}{\underset{i,j}{\sum }\exp\left(e_{i,j}\right)}$ Weighted features: $F_{attn}=\underset{t}{\sum }\underset{f}{\sum }\alpha_{t,f}{\left[F_{CNN};F_{LSTM}\right]}_{t,f}$ Prediction output: $\hat{y}=W_{o}\cdot F_{attn}+b_{o}$	Epochs [100~500] Batch size [1~64] Optimizer = [Adam] Learing rate [0.0001~0.01] LSTM hidden units [32, 64, 128] CNN hidden units [16, 32, 64] Attention heads [1, 2, 4] Dropout rate [0.1~0.5]

SARIMA, seasonal autoregressive integrated moving average; SVR, support vector regression; GRU, gated recurrent unit; CNN, convolutional neural network; LSTM, long short-term memory; AIC, Akaike information criterion.

## 3 Results

### 3.1 Epidemiological characteristics of AIDS incidence in China

**Figure 3:**
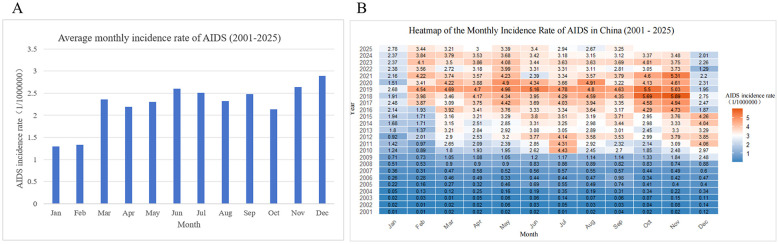
Seasonal and temporal patterns of AIDS incidence in China. **(A)** Bar chart of average monthly incidence rates (per 1,000,000 population) averaged over the study period (2001–2025), showing seasonal peaks during winter months. **(B)** Heatmap of monthly incidence rates (per 1,000,000 population) from 2001 to 2025, with warmer colors indicating higher incidence, illustrating the long-term upward trend and consistent seasonal fluctuations.

Analysis of monthly AIDS incidence from January 2001 to October 2025 (Figure 3A) revealed that monthly AIDS incidence in China was predominantly elevated during winter months, whereas lower rates were concentrated in spring, with a secondary peak occurring during June–July, forming a bimodal seasonal distribution. Heatmap visualization (Figure 3B) intuitively demonstrated a continuous upward trend in incidence throughout 2001–2025, with relatively gradual growth from 2001 to 2009, accelerated increase during 2010–2020, and subsequent maintenance at elevated levels with minor fluctuations after 2020. October through December consistently represented deep-red high-incidence periods each year. Overall, these findings clearly indicate a significant upward trend in monthly AIDS incidence in China, characterized by stable winter peaks, and the recent growth trend has not been effectively curbed. Notably, pronounced fluctuations in incidence around 2020 suggest potential abnormal observations caused by external factor interference. To ensure that models learn authentic epidemiological patterns rather than data noise, this study implemented formal seasonal outlier detection (S-H-ESD) prior to model training. The S-H-ESD algorithm identified several potential outliers, primarily including December 2019–January 2020 (early COVID-19 pandemic, reduced active testing leading to artificially decreased reported cases) and October–December 2020 (post-pandemic compensatory testing leading to anomalous rebound in incidence). These outliers were flagged prior to model training but retained within the dataset to reflect real epidemiological fluctuations.

### 3.2 Comparative performance evaluation of predictive models

We systematically evaluated 10 predictive models: conventional time series (ARIMA), traditional machine learning (SVR and Random Forest), unimodal deep learning (GRU, CNN, and LSTM), and hybrid deep learning architectures (GRU-LSTM serial fusion, LSTM-CNN parallel fusion, LSTM-GRU-Attention, and LSTM-CNN-Attention). Monthly AIDS incidence in China exhibited distinct seasonal patterns. We therefore employed a Seasonal Autoregressive Integrated Moving Average (SARIMA) model.Detailed construction processes and results for the SARIMA model are provided in Supplementary material Attachment 1 (S1).

**Figure 4:**
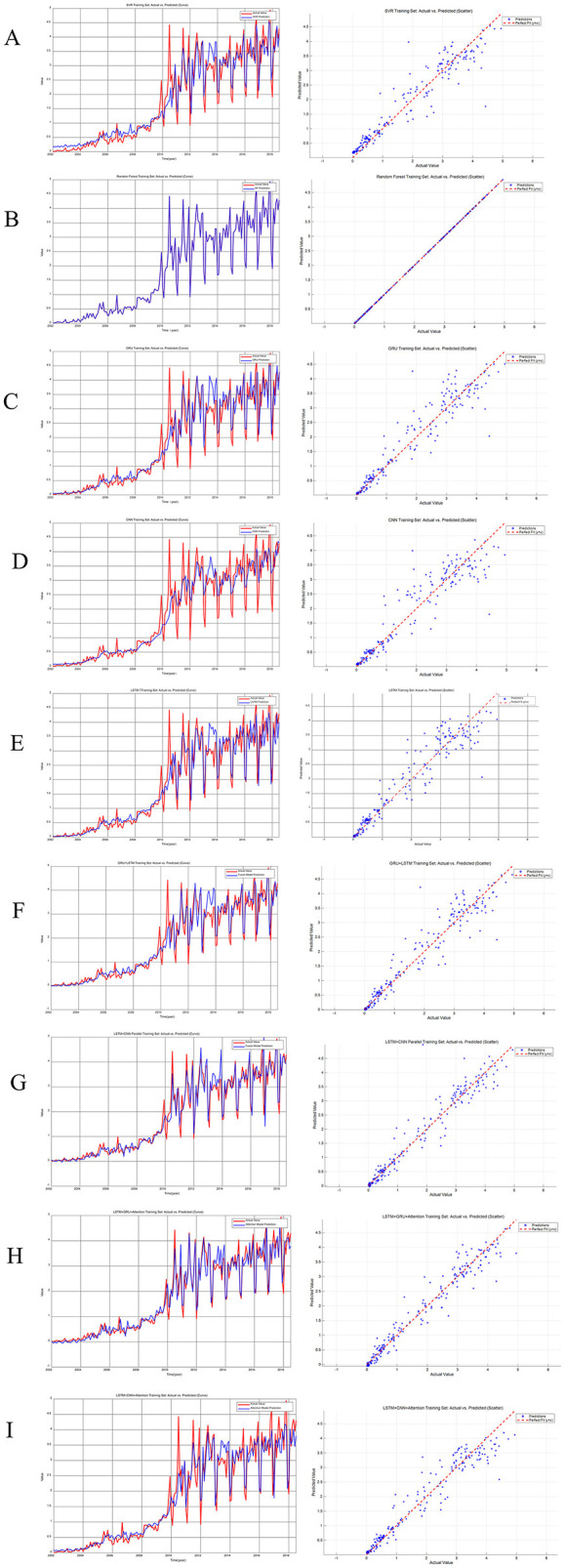
Fitting performance of nine predictive models on the training set. Left side: Time-series plots comparing model predictions (blue curves) against observed values (red curves). Right side: Scatter plots of predicted vs. actual values (dashed line indicates perfect fit). **(A)** Support vector regression (SVR); **(B)** Random forest; **(C)** Gated recurrent unit (GRU); **(D)** Convolutional neural network (CNN); **(E)** Long short-term memory (LSTM); **(F)** GRU-LSTM serial fusion; **(G)** LST-CNN parallel fusion; **(H)** LSTM-GRU-Attention; **(I)** LSTM-CNN-Attention.

To intuitively evaluate the fitting performance of each model on the training set, this study generated prediction curve–actual observation sequence comparison plots and prediction–actual value scatter plots for nine learning models (Figures 4A–I). Regarding time-series fitting, the Random Forest model demonstrated prediction curves almost completely overlapping with observed incidence trajectories, achieving the optimal goodness-of-fit; the LSTM-CNN parallel fusion model ranked second, with overall trends closely aligned. Regarding scatter distribution, Random Forest data points clustered densely along the diagonal, indicating minimal prediction residuals; LSTM-CNN data points also showed favorable concentration trends; other models displayed relatively dispersed scatter patterns, suggesting certain prediction deviations. However, high goodness-of-fit on the training set does not equate to favorable generalization capability. Combined with quantitative evaluation on the validation set (Table 2), the Random Forest model exhibited a pronounced overfitting tendency, whereas the LSTM-CNN parallel fusion model achieved a relatively better balance between fitting precision and generalization stability and was selected as the relatively better-performing predictive model within this dataset.

**Table 2:** The results of 10 models on the validation set.

Model name	*R* ^2^	MAE	MAPE (%)
LSTM+CNN	0.605	0.456	14.690
SVR	0.561	0.497	15.750
GRU	0.493	0.555	16.950
LSTM+CNN+attention	0.477	0.523	15.640
GRU+LSTM	0.479	0.554	17.400
CNN	0.380	0.571	19.490
LSTM	0.335	0.599	17.930
LSTM+GRU+attention	0.335	0.568	18.070
Random forest	0.198	0.678	19.890
SARIMA	0.240	21.872	65.800

*R*^2^, coefficient of determination; MAE, mean absolute error; MAPE, mean absolute percentage error; SARIMA, seasonal autoregressive integrated moving average; SVR, support vector regression; GRU, gated recurrent unit; CNN, convolutional neural network; LSTM, long short-term memory.

**Figure 5:**
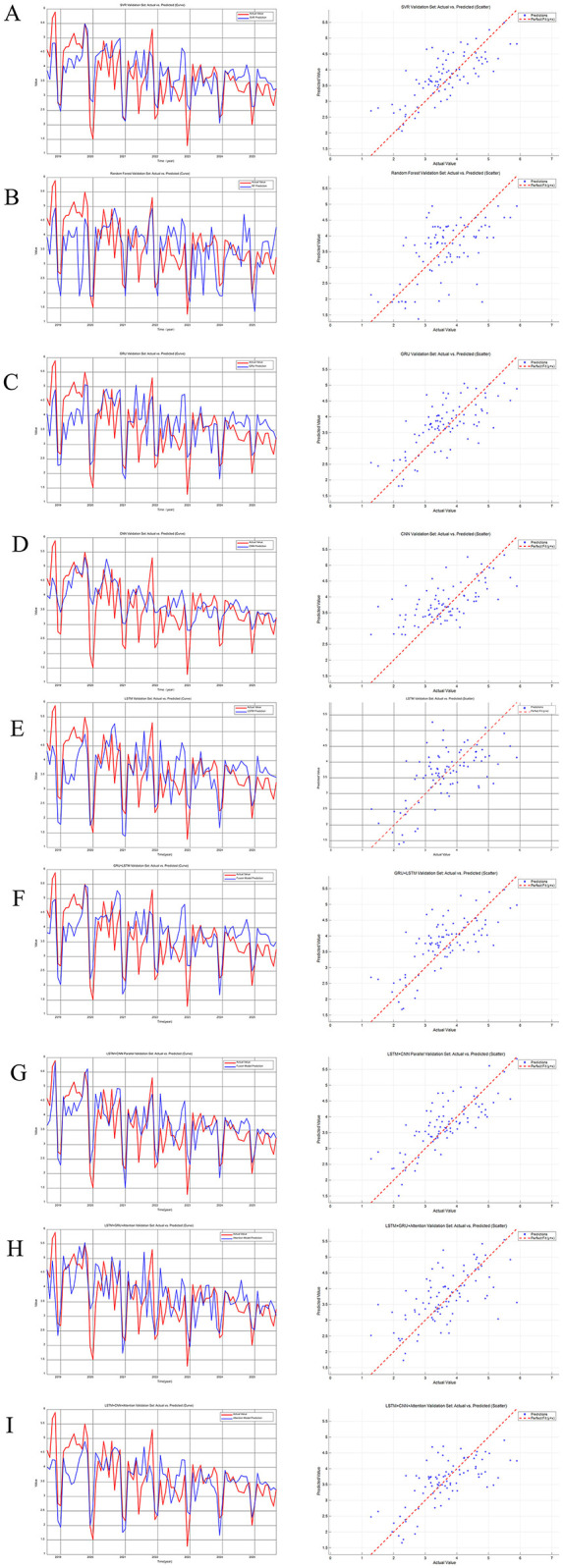
Generalization performance of nine predictive models on the validation set. Left side: Time-series plots comparing model predictions (blue curves) against observed values (red curves). Right side: Scatter plots of predicted vs. actual values (dashed line indicates perfect fit). **(A)** Support vector regression (SVR); **(B)** Random forest; **(C)** Gated recurrent unit (GRU); **(D)** Convolutional neural network (CNN); **(E)** Long short-term memory (LSTM); **(F)** GRU-LSTM serial fusion; **(G)** LSTM-CNN parallel fusion; **(H)** LSTM-GRU-Attention; **(I)** LSTM-CNN-Attention.

Furthermore, we generated prediction time-series, actual time-series, and ‘prediction–actual' scatter plots for the models on the validation set (Figures 5A–I). Results indicated differences in generalization performance across models on the validation set: the LSTM-CNN parallel fusion model demonstrated relatively superior generalization performance within this dataset, with prediction curves showing better alignment with actual trajectories compared to other models. Traditional machine learning models, particularly Random Forest, showed relatively weak generalization performance; combined with training set results, Random Forest exhibited pronounced overfitting on the training set. These results indicate that the LSTM-CNN parallel fusion model achieved relatively superior generalization on the validation set in this study; among the 10 algorithms, its R^2^ = 0.605, MAE = 0.456, MAPE = 14.690%, representing the maximum R^2^ and minimum MAE and MAPE values.

The results of 10 models on the validation set (Table 2) demonstrated that the LSTM-CNN model achieved relatively superior predictive performance on the validation set in this study, with R^2^ = 0.605, MAE = 0.456, MAPE = 14.690%, representing the maximum R^2^ and minimum MAE and MAPE values. Although Random Forest exhibited excellent fitting on the training set, it demonstrated extremely poor generalization on the validation set, with severe overfitting. Some fusion models (e.g., LSTM-CNN-Attention) performed slightly worse than the LSTM-CNN model. In this study, the SARIMA model exhibited the poorest generalization capability. Detailed construction processes and results for the 10 models evaluated in this study are provided in Supplementary Material Attachment 1 (S1–S10).

### 3.3 Characteristics of prediction error distributions

**Figure 6:**
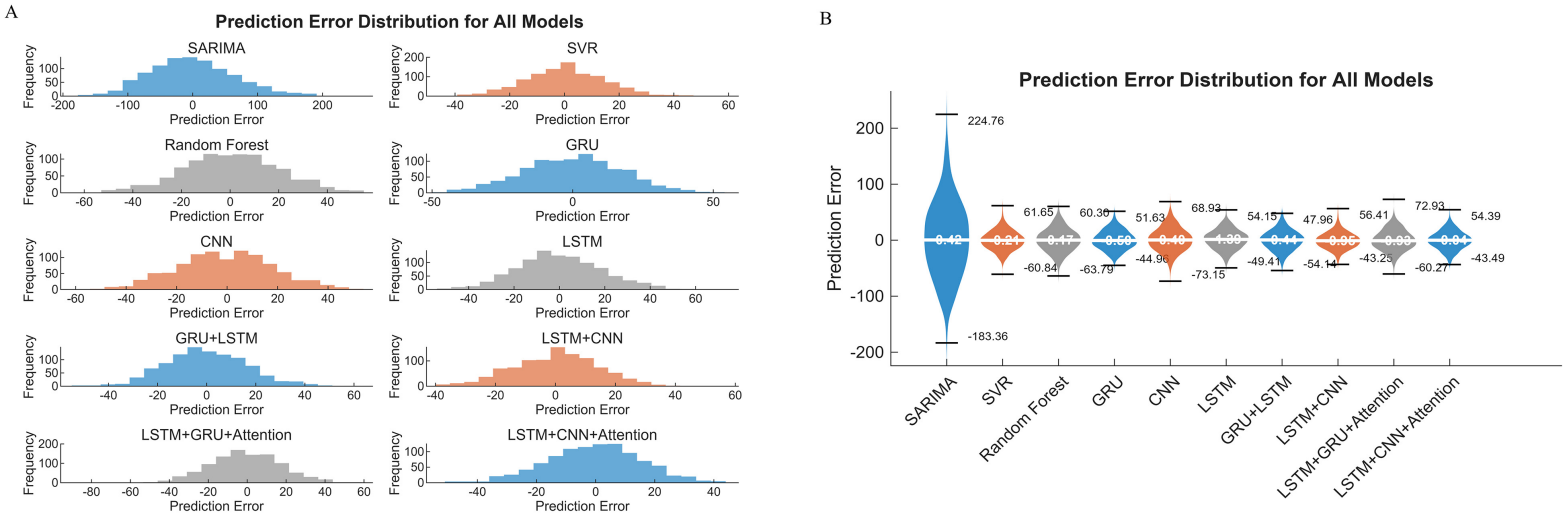
Distribution characteristics of prediction errors across 10 models. **(A)** Histograms of prediction errors (difference between predicted and actual incidence rates), with the horizontal axis representing prediction error and the vertical axis representing frequency. **(B)** Violin-box plots of prediction errors, where violin shapes reflect the density distribution of errors, and box plots indicate the range (whiskers) and median (center line) of errors. SARIMA, seasonal autoregressive integrated moving average; SVR, support vector regression; GRU, gated recurrent unit; CNN, convolutional neural network; LSTM, long short-term memory.

To intuitively analyze the stability and precision of prediction results across models, this study examined prediction error histograms (Figure 6A) and violin-box plots (Figure 6B), comparing model performance from dimensions including distribution concentration and fluctuation range. As shown in the prediction error histograms (Figure 6A), the SARIMA model exhibited error ranges spanning −200 to 200, with dispersed frequency distributions and no distinct peaks, indicating substantial volatility and the poorest stability. The LSTM-CNN model demonstrated error concentration within the −40 to 40 interval, with peaks adjacent to zero and the highest frequency, representing the model with errors closest to true values and the most compact distribution among all models. As shown in the prediction error violin-box plots (Figure 6B), SARIMA exhibited the widest violin shape, indicating highly dispersed error distribution; LSTM-CNN showed a relatively narrower violin shape compared to other models, with extreme values ranging from −43.25 to 56.41, representing a relatively smaller fluctuation range and comparatively better error concentration among the evaluated models. These results further confirm that, in this study, the LSTM-CNN fusion model achieved superior performance in error concentration, stability, and precision, whereas the traditional time-series model (SARIMA) exhibited large error fluctuations and poor stability, rendering it the least suitable for the AIDS incidence forecasting task in this study.

### 3.4 Forecasting results of the LSTM-CNN parallel fusion model

**Figure 7:**
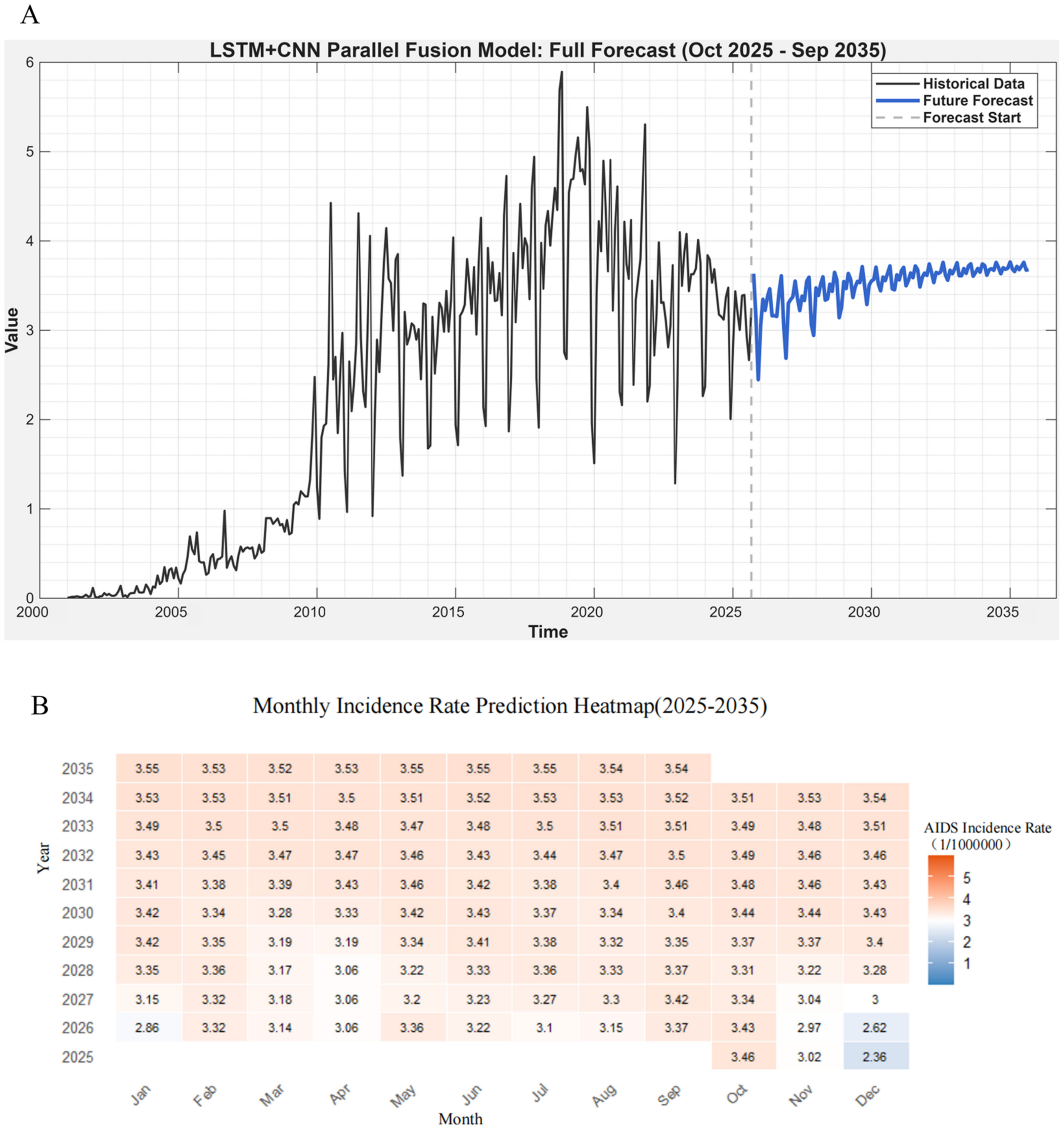
Forecasting Results of the LSTM-CNN Parallel Fusion Model. **(A)** Time series projection (2001–2035). The black line represents historically observed data (January 2001–September 2025), and the blue line represents the future forecast (October 2025–September 2035). The dashed line indicates the forecast start point. **(B)** Heatmap of forecasted monthly AIDS incidence in China (October 2025–September 2035), with color intensity indicating incidence rate (per 1,000,000).

Based on the LSTM-CNN parallel fusion model, which exhibited relatively superior validation performance in this study, we projected monthly AIDS incidence over the 10-year period from October 2025 to September 2035 (Figure 7). The time-series projection of monthly AIDS incidence over the next decade (Figure 7A) revealed a ‘slowly rising trend with fluctuations'; incidence rates are expected to maintain relatively elevated levels after 2030, with persistent intra-annual seasonal fluctuation characteristics (e.g., year-end high-incidence peaks) throughout the forecast period. The projected monthly incidence heatmap over the next decade (Figure 7B) illustrated consistent annual seasonal fluctuations in incidence throughout the forecast period (e.g., universal increases during November–December), with AIDS incidence in China projected to rise from 2025 to 2030, after which the upward trajectory is expected to decelerate while remaining elevated. By September 2035, the projected monthly incidence rate is 3.54 per 1,000,000 population (95% Cl: 3.17–3.90 per 1,000,000 population). The 95% confidence intervals for the prediction results are detailed in Supplementary Material Attachment 2.

## 4 Discussion

This study collected monthly AIDS incidence data from China spanning January 2001 to September 2025, and systematically analyzed the performance of 10 models—including SARIMA, SVR, LSTM, and LSTM-CNN parallel fusion—in forecasting monthly AIDS incidence in China. Our results demonstrate that the LSTM-CNN parallel fusion model achieved relatively superior generalization on the validation set within this dataset. Error distribution analysis further indicated that this model exhibited comparatively better performance in concentration, stability, and precision of prediction errors relative to other evaluated models. These findings provide crucial evidence for model selection in early warning systems and the formulation of prevention and control strategies.

Relative to global averages, China is classified as a low-prevalence region for HIV/AIDS; however, the absolute number of people living with HIV/AIDS remains substantial given its large population base. As of December 31, 2024, approximately 1.355 million cases had been reported nationwide. While global AIDS incidence has declined markedly in recent years, the epidemic in China has entered a plateau phase. This study reveals significant seasonal fluctuations in monthly AIDS incidence, with peak rates occurring in winter, consistent with previous reports and investigations in Xinjiang (29, 31). This seasonal pattern likely reflects increased healthcare-seeking behavior during winter months for influenza and other respiratory infections, combined with higher rates of HIV testing during year-end health examinations, leading to increased reporting rates and indicating a potentially substantial reservoir of undiagnosed infections. However, it must be specifically noted that this seasonal pattern may also partially reflect reporting bias: year-end health examinations and active testing during winter may lead to elevated reported incidence rates rather than true increases in transmission rates. Recent years have witnessed a bimodal age distribution among key affected populations, with incidence rising among both young and older population (32–34). Enhanced screening and precisely targeted interventions for these demographic groups are imperative to sustain prevention and control achievements. Notably, pronounced data fluctuations around 2020 are closely related to the impact of the COVID-19 pandemic on HIV/AIDS case identification in China. Zhao et al. (35) demonstrated through interrupted time-series analysis that during the early pandemic period (January–March 2020), HIV/AIDS case reporting declined significantly due to disruptions in surveillance and testing services; as pandemic control measures were implemented and services restored, compensatory reporting increases emerged during the latter half of 2020. The S-H-ESD outlier detection in this study also identified data anomalies during this period. This external shock highlights the fundamental limitations of purely endogenous time-series models: they cannot anticipate or adapt to such sudden public health emergencies, leading to prediction biases.

A key finding is the superior performance of the LSTM-CNN parallel fusion model, highlighting the potential of hybrid architectures to enhance representational capacity. This aligns with findings from multiple studies. Brazilian scholars have demonstrated that CNN-LSTM fusion models significantly outperform classical approaches in predicting tuberculosis/HIV co-infection incidence (36). In this study, the LSTM-CNN parallel fusion model achieved the highest R^2^ and lowest error rates on the validation set, further validating its capability to capture long-term dependencies and nonlinear trends. The unique gating mechanism of LSTM enables effective learning of long-term dependencies, excelling at capturing underlying temporal trends behind these characteristics while enhancing sensitivity to peak incidence period identification, and mitigating vanishing or exploding gradient problems inherent in conventional recurrent neural networks (RNNs) (37). Concurrently, CNN modules efficiently extract local patterns and short-term fluctuations from the data (38). Similar hybrid strategies have been validated in forecasting other diseases. For instance, hybrid LSTM-ARIMA models for predicting pediatric AIDS incidence in East Asia (21) and SARIMA-LSTM models for influenza forecasting (39) have both demonstrated superior performance compared with single-model approaches. This ‘complementary advantages' design philosophy represents an effective approach to addressing the mixture of linear and nonlinear components inherent in epidemiological data (25). These findings demonstrate the potential effectiveness of deep learning models in capturing complex patterns within such datasets.

Compared with other models in this study, the LSTM-CNN parallel fusion model demonstrated relatively superior performance. The Random Forest model exhibited severe overfitting, characterized by excellent training-set performance but significant degradation on the validation set. This phenomenon, marked by high training accuracy coupled with poor generalization, is commonly observed in traditional machine learning applied to high-volatility time-series data. In contrast, traditional statistical models such as SARIMA demonstrated inferior predictive performance relative to deep learning architectures, consistent with previous findings. Specifically, single ARIMA models exhibit limited generalization capacity when addressing sudden fluctuations in forecasting AIDS incidence and mortality in China (40). Additionally, ARIMA models exhibited degraded performance during public health emergencies (e.g., the COVID-19 pandemic) owing to their inability to effectively model heteroscedasticity (i.e., volatility clustering) (35). These limitations underscore the inherent constraints of conventional approaches in handling high-volatility, non-stationary time-series data.

We also observed that single deep learning models (e.g., GRU, CNN) and the GRU-LSTM hybrid were less accurate than the LSTM-CNN parallel fusion on the validation set, possibly due to excessive architectural complexity, limited training data, or suboptimal hyperparameter tuning. The GRU model, a simplified LSTM variant, performed worse than the LSTM-CNN fusion in this study, possibly due to dataset complexity and sequence length variability, consistent with documented performance variations across applications (39). It must be emphasized that the above comparative conclusions are limited to the dataset and experimental settings of this study. Dataset characteristics (e.g., sequence length, noise level, seasonal intensity) may alter model rankings. Additionally, we incorporated attention mechanisms into LSTM-CNN-Attention and LSTM-GRU-Attention architectures, wherein the attention mechanism is responsible for intelligently ‘focusing on' the most important parts of historical sequences when generating predictions. However, neither attention-enhanced model outperformed the LSTM-CNN parallel fusion, likely reflecting over-parameterization. This may be attributable to the fact that this dataset comprises a single disease, with overly simplistic parameter settings, relatively limited time-series length (297 months), and feature dimensionality (univariate), whereas the advantages of attention mechanisms are more readily manifested in complex, high-dimensional data. Therefore, future research may consider introducing additional regularization strategies or external covariates (e.g., policy interventions) to enhance model robustness.

Based on the LSTM-CNN parallel fusion model, which demonstrated optimal performance in this study, we projected monthly HIV incidence in China over the next decade. Projections indicate that AIDS incidence in China will increase from 2025 to 2030, followed by a deceleration in growth; however, incidence will remain elevated. Monthly incidence continues to exhibit annual seasonal fluctuations, peaking during winter months. These projections suggest that achieving the United Nations goal of ending AIDS by 2030 remains challenging (41). Nevertheless, these projections provide valuable insights for long-term prevention strategies, indicating a need for sustained, intensive interventions targeting key populations.

All models in this study were constructed using historical incidence data only, representing an endogenous analysis framework. However, AIDS incidence is significantly influenced by external factors such as fluctuations in HIV testing volume (e.g., active case-finding effects resulting from expanded testing campaigns), preventive interventions (e.g., PrEP/PEP promotion), holiday population mobility and social behavior changes, and health education outreach coverage. These exogenous variables constitute ‘epidemiological noise,' making it difficult for purely endogenous models to fully capture true dynamics. Therefore, model predictions are essentially ‘scenario-free statistical extrapolation' rather than ‘multifactorial epidemiological projection.' Long-term projections (to 2035) do not incorporate potential impacts of future policy changes (e.g., universal PrEP coverage, novel testing technology promotion) on incidence trajectories, and results should be interpreted solely as ‘baseline trend reference.'

Accurate forecasting is fundamental to effective prevention and control. In this study, we confirmed that the LSTM-CNN parallel fusion model can serve as a reliable early warning tool. The United Nations has set ambitious targets to end the AIDS epidemic by 2030 through its Political Declaration on HIV and AIDS (41). Achieving these goals requires data-driven, precision prevention and control strategies. As Xu et al. emphasize, a central pillar of China's AIDS control strategy involves expanding testing and facilitating early diagnosis (42). High-performance predictive models enable public health authorities to anticipate epidemic peaks and high-burden regions, thereby optimizing testing resources and antiretroviral drug allocation in advance, and facilitating targeted interventions for high-risk populations. This directly contributes to achieving the ‘three 95s' targets: diagnostic coverage, treatment coverage, and viral suppression (41). Analysis of AIDS incidence characteristics in China reveals a non-stationary time series characterized by ‘long-term fluctuating increases,' with significant seasonality observable. The continued upward trend in recent years, which has not been effectively curbed, indicates that current HIV strategies and interventions remain insufficient for reducing disease incidence. This underscores the urgency of reviewing and strengthening prevention, diagnostic, and therapeutic measures. Seasonality presents distinct challenges for AIDS control, necessitating intensified, targeted interventions for high-risk populations during winter months. Given these challenges, predictive models play a pivotal role in AIDS control and management.

From this perspective, this study examined four methodological approaches for infectious disease forecasting. The first category comprises classical statistical models (SARIMA); the second category includes conventional machine learning algorithms (SVR and Random Forest); the third category encompasses deep learning models (LSTM, CNN, and GRU); and the fourth category involves four hybrid fusion models built upon the aforementioned models (GRU-LSTM serial fusion, LSTM-CNN parallel fusion, LSTM-GRU-Attention, and LSTM-CNN-Attention). Although classical statistical models have achieved relative success in infectious disease forecasting, they encounter substantial challenges in capturing nonlinear relationships within time series. Deep learning offers the distinct advantage of capturing complex, nonlinear patterns without explicit specification of input-output functional forms (43). Within our framework, deep learning models, particularly the LSTM-CNN parallel fusion, demonstrated superior predictive performance with lower error rates than SARIMA and SVR, indicating greater efficiency in capturing complex epidemiological patterns. Notably, this study features extended temporal coverage and comprehensive benchmarking of 10 models, providing a more robust framework for comparative analysis of diverse epidemiological data.

Although this study achieved preliminary results in model comparison, several limitations remain. All models were constructed using historical incidence data only, without incorporating external variables such as socioeconomic factors, population mobility, or control policies. Integrating these multidimensional determinants would facilitate a more comprehensive understanding of drivers underlying AIDS trends and enhance predictive accuracy. Additionally, deep learning architectures require substantial computational resources, and hyperparameter tuning processes have not been fully systematized, potentially affecting model stability. Furthermore, this study was strictly limited to univariate time-series forecasting, without exploring multivariate frameworks incorporating covariates such as testing volume or ART coverage. Although this methodological choice ensured fair comparison among models, it indeed weakened epidemiological interpretability and policy relevance. Moreover, given the moderate predictive performance of the model (R^2^ = 0.605), the inherent limitations of the purely endogenous analysis framework, and the structural constraints of not incorporating external predictor variables, prediction results should be interpreted solely as statistical trend references. In future research, we will construct multivariate predictive frameworks incorporating covariates such as testing volume, PrEP/PEP distribution, and policy indicators, and conduct gender/age-group stratified analyses to enhance the epidemiological interpretability and predictive robustness of the model. It should be noted that this study is based on a single national-level incidence series, Model rankings are based solely on point estimates of predictive metrics (R^2^, MAE, MAPE), and no formal statistical tests (e.g., Diebold-Mariano test) were conducted to assess whether performance differences between models reached statistical significance. Consequently, the “relatively superior” performance of the LSTM-CNN model refers to metric-based ranking within this specific dataset. In future research, more diversified evaluation metrics (e.g., NRMSE, WAPE, sMAPE) will be incorporated, and optimization algorithm improvements such as the PSO-RDV IW technique from Aribe Jr (44) will be adopted to introduce metaheuristic methods (e.g., particle swarm optimization, genetic algorithms) into the hyperparameter tuning process of the LSTM-CNN model, thereby further enhancing model convergence efficiency and prediction stability. Nevertheless, within the conditions of this dataset, this study observed relatively superior validation performance of the LSTM-CNN parallel fusion model for forecasting monthly AIDS incidence in China. Particularly in the context of major infectious disease outbreaks where multidimensional data are difficult to obtain, the LSTM-CNN parallel fusion model constructed in this study may serve as a preliminary methodological exploration within univariate time-series forecasting. This model may provide an auxiliary analytical tool for understanding the temporal dynamics of AIDS epidemics and exploratory data references for public health surveillance. Furthermore, the predictive framework established in this study may offer limited methodological insights for time-series forecasting research on other infectious diseases at the methodological level; however, its transferability awaits validation across additional diseases and datasets.

In summary, this study compared the predictive performance of 10 different time-series analysis and deep learning models for forecasting monthly AIDS incidence in China. Within this dataset and methodological framework, the LSTM-CNN parallel fusion model exhibited relatively superior validation performance. These findings provide an effective predictive tool and novel methodological insights for time-series forecasting in epidemiology.

## 5 Conclusions

This study comparatively evaluated the predictive performance of 10 models—SARIMA, SVR, Random Forest, GRU, CNN, LSTM, GRU-LSTM serial fusion, LSTM-CNN parallel fusion, LSTM-GRU-Attention, and LSTM-CNN-Attention—within a univariate time-series forecasting framework. Under the conditions of this specific dataset, the LSTM-CNN parallel fusion model exhibited relatively superior validation performance for forecasting monthly AIDS incidence in China, suggesting potential applicability of hybrid deep learning architectures for infectious disease time-series forecasting. Projections from this model indicate that monthly AIDS incidence in China may follow an upward trajectory with fluctuations over the next decade.

## Data Availability

The datasets presented in this study can be found in online repositories. The names of the repository/repositories and accession number(s) can be found below: National Notifiable Disease Reporting System (NNDRS), Chinese Center for Disease Control and Prevention (https://www.chinacdc.cn/jksj/jksj01/).
