# Supplementary material for: Forecasting monthly AIDS incidence in China via LSTM-CNN parallel fusion: a comparative study of 10 predictive models
Source: Front Public Health. 2026 Jul 29;14:1873637. doi: 10.3389/fpubh.2026.1873637 (PMC13461732; doi:10.3389/fpubh.2026.1873637)
Supplement: Supplementary file 1 [file Supplementary_file_1.docx]

**Supplementary Material（attachment 1）**

**Forecasting Monthly AIDS Incidence in China via LSTM-CNN Parallel Fusion: A Comparative Study of Ten Predictive Models**

Chengcheng Li^1†^ , Jinfeng Li^2†^ , Shifeng Pang^3†^ , Chao Rong^1^, Ximan Ye^2^, Xiaojie Lao^4*^, Maowei Chen^2*^

^1^ Humanities and Management School, Zhejiang Chinese Medical University, Hangzhou, Zhejiang, China,

^2^ Department of Infectious Diseases, Wuming Hospital Affiliated to Guangxi Medical University, Nanning, Guangxi, China

^3^ Department of Cardiology, Minzu Hospital of Guangxi Zhuang Autonomous Region, Nanning, Guangxi, China

^4^ Department of Cardiology, Sir Run Run Shaw Hospital, Zhejiang University School of Medicine, Hangzhou, Zhejiang, China

**^†^** Chengcheng Li, Jinfeng Li, and Shifeng Pang contributed equally to this study

*** Correspondence:**

Xiaojie Lao

No. 3, Qingchun East Road, Shangcheng District, Hangzhou, Zhejiang Province

Email: xiaojielao@126.com (X. Lao), ORCID iD: 0009-0009-4675-6480

Maowei Chen

No. 26 Yongning Road, Wuming District, Nanning, Guangxi, China

Email: chenmaowei2008@163.com (M. Chen), ORCID iD: 0000-0001-8673-4328

Total pages: 22 (including cover page)

**S1. Seasonal Autoregressive Integrated Moving Average (SARIMA) Model: Construction and Results**

**S1.1 SARIMA Model Construction**

Time series analysis was performed using the Seasonal Autoregressive Integrated Moving Average (SARIMA) model, which captures non-stationary temporal patterns through the combination of autoregressive (AR), differencing (I), and moving average (MA) components, augmented by seasonal parameters to account for periodic fluctuations (1). Model identification was conducted using the Autocorrelation Function (ACF) and Partial Autocorrelation Function (PACF) to determine model orders, with model selection guided by the Akaike Information Criterion (AIC). Parameter estimation was performed via maximum likelihood estimation.

The SARIMA model was implemented on the R 4.5.0 platform, with a seasonal period of s = 12. First, the Augmented Dickey-Fuller (ADF) unit root test was applied to assess series stationarity; non-stationary sequences were differenced accordingly. Subsequently, the ACF and PACF plots of the differenced series were examined, and the AIC criterion was used to determine the non-seasonal orders (p, d, q) and seasonal orders (P, D, Q).

The SARIMA model is expressed in Equation (1):

$\Phi_{P}(B^{s})\phi_{p}(B)\nabla^{d}\nabla_{s}^{D}X_{t}=\Theta_{Q}(B^{s})\theta_{q}(B)\varepsilon_{t}$	(1)

In formula (1) : $X_{t}$denotes the time series value; B represents the backshift operator, where $B^{k}X_{t}=X_{t-k}$; $\nabla=(1-B)$ is the non-seasonal differencing operator; $\nabla_{s}=(1-B^{s})$ is the seasonal differencing operator; s is the seasonal period ($s=12$ in this study); $\phi_{p}(B)=1-\sum_{i=1}^{p} \phi_{i}B^{i}$ and $\theta_{q}(B)=1+\sum_{j=1}^{q} \theta_{j}B^{j}$ are the non-seasonal autoregressive and moving average polynomials, respectively; $\Phi_{P}(B^{s})=1-\sum_{i=1}^{P} \Phi_{i}B^{\mathrm{is}}$ and $\Theta_{Q}(B^{s})=1+\sum_{j=1}^{Q} \Theta_{j}B^{\mathrm{js}}$ are the seasonal autoregressive and moving average polynomials, respectively; and ε_t is the random error term, assumed to follow a white noise distribution, i.e.,$\varepsilon_{t}\sim WN(0,\sigma^{2})$.

**S1.2 SARIMA Model Results**

Descriptive analysis was performed on the raw monthly HIV/AIDS incidence data in China, and a time series plot of the raw data from January 2001 to September 2025 was generated (Figure S1A). The monthly HIV/AIDS incidence data in China exhibited a non-stationary characteristic of long-term fluctuating increase. First, the ADF unit root test indicated the presence of a unit root (P > 0.1); therefore, first-order non-seasonal differencing (d = 1) was applied. The differenced series satisfied the stationarity requirement (P < 0.05). Examination of the ACF and PACF plots of the first-differenced series revealed that the ACF truncated at lag 4 and the PACF showed no clear truncation pattern; thus, the non-seasonal moving average order was determined as q = 4 and the autoregressive order as p = 0. Meanwhile, the incidence time series curve (Figure S1A) indicated a 12-month seasonal cycle (annual end-of-year peak pattern). Given the periodic seasonal characteristics of the data, the basic ARIMA model was insufficient to explain the seasonal fluctuations; therefore, within the original Box-Jenkins modeling framework, seasonal parameters were added to extend ARIMA to SARIMA. Both models followed the same comprehensive modeling procedure, including stationarity testing, AIC-BIC criterion-based order selection, and residual white noise testing. Seasonal ADF testing indicated the presence of a seasonal unit root (P > 0.1); hence, first-order seasonal differencing (D = 1) was performed. Further examination of the seasonal ACF/PACF plots after seasonal differencing revealed that the seasonal ACF truncated at lag 12 and the seasonal PACF showed no clear truncation; accordingly, the seasonal moving average order was determined as Q = 1 and the seasonal autoregressive order as P = 0. The final model was identified as SARIMA(0,1,4)(0,1,1)₁₂.

After identifying SARIMA(0,1,4)(0,1,1)₁₂, Figure S1B illustrates that the SARIMA(0,1,4)(0,1,1)₁₂ model achieved stationarity through differencing of the non-stationary time series. The model training set R² = 0.914 indicated a certain degree of fitting capability. Ultimately, the validation set metrics for the SARIMA(0,1,4)(0,1,1)₁₂ model were: R² = 0.240, MAE = 21.872, and MAPE = 65.800%.

To evaluate the adequacy of the model fit, the residual autocorrelation function (ACF) plot (Figure S1C) and residual distribution histogram (Figure S1D) were generated. Figure S1C shows that the autocorrelation coefficients at several lag orders exceeded the confidence interval; the Ljung-Box white noise test yielded p < 0.05, rejecting the null hypothesis that the residuals follow a white noise process, indicating residual autocorrelation—specifically, the pattern of "historical values influencing current values" remained uncaptured by the model. The residual distribution histogram (Figure S1d) did not exhibit an ideal normal distribution (peak shifted left, asymmetric tails), further confirming insufficient model fitting and the presence of non-random deviations in the residuals. Based on the SARIMA(0,1,4)(0,1,1)₁₂ model, a comparison plot of predicted versus actual values on the training set was produced (Figure S1E) to visually demonstrate predictive performance. The results showed low agreement between predicted (blue) and actual (red) values; the predicted curve failed to track the sharp fluctuations of the actual data, only broadly fitting the long-term trend, with substantial local errors.

Collectively, the SARIMA validation set MAPE of 65.8% was markedly higher than that of other models, revealing the structural limitations of traditional statistical models in addressing complex public health time series: (1) SARIMA assumes a linear data-generating mechanism, whereas HIV/AIDS incidence is driven by multiple nonlinear factors; (2) SARIMA is sensitive to structural breaks; (3) SARIMA relies solely on the series' own lagged information without incorporating external covariates. The large discrepancy between the training set R² = 0.914 and the validation set R² = 0.2400 suggests severe overfitting risk. Moreover, the SARIMA residuals did not satisfy the ideal conditions of "stationarity, no autocorrelation, and normality," indicating that the model failed to adequately accommodate the complex temporal characteristics of HIV incidence.

**Figure S1. Fitting and diagnostic results of the SARIMA(0,1,4)(0,1,1)_12_ model.**

(A) Original time series of monthly AIDS incidence in China (January 2001–September 2025). (B) Time-series plot of residuals. (C) Autocorrelation Function (ACF) plot of residuals. (D) Distribution histogram of residuals with normal distribution fit overlaid.. (E) Comparison of actual observed values (red curve) versus model predictions (blue curve) on the validation set (R² = 0.240, MAPE = 21.872, MAE = 0.658).

**S2. Support Vector Regression (SVR) Model: Construction and Results**

**S2.1 SVR Model Construction**

Support Vector Regression (SVR) achieves continuous value prediction by identifying the optimal hyperplane. The model maximizes the margin between support vectors while minimizing regression errors, subject to an error tolerance ε (2). Given training data$\{(x_{i},y_{i}){\}}_{i=1}^{n}$, the regression function is expressed in Equation (2), and the constraints are defined in Equation (3), where$\phi(x)$ is the kernel-induced feature mapping, $\omega$ is the weight vector, $b$ is the bias term, and $\xi_{i}$ and $\xi_{i}^{*}$ are slack variables. The Radial Basis Function (RBF) was adopted as the kernel function, and the regularization parameter $C$ and kernel parameter $\gamma$ were optimized via 5-fold cross-validation.

The SVR model was implemented on the MATLAB R2023b platform (MathWorks, USA) using the fitrsvm function from the Statistics and Machine Learning Toolbox. The time step k was determined via grid search over [3, 6, 12, 24]. All evaluation metrics were computed on Z-score standardized data to ensure fair comparison across models.

**Regression function:**

$f(x)=\omega^{T}\phi(x)+b$	(2)

**Subject to constraints:**

$\left\{ \begin{matrix} y_{i}-\omega^{T}\phi(x_{i})-b\leq\varepsilon+\xi_{i} \\ \omega^{T}\phi(x_{i})+b-y_{i}\leq\varepsilon+\xi_{i}^{*} \\ \xi_{i},\xi_{i}^{*}\geq0 \end{matrix} \right.$	(3)

In formula (2-3): $\phi(x)$ is the kernel-induced feature mapping,$\omega$ is the weight vector, and$b$ is the bias term; $\xi_{i}$and$\xi_{i}^{*}$ are slack variables, and $\varepsilon$ is the error tolerance.

**S2.2 SVR Model Results**

Support Vector Regression (SVR) is a supervised learning algorithm based on the principle of structural risk minimization. By introducing a kernel function, SVR maps low-dimensional nonlinear problems into a high-dimensional feature space for regression solution. Leveraging the favorable statistical properties and generalization performance of SVR under small-sample conditions, this study adopted the Radial Basis Function (RBF) as the kernel function and utilized its excellent nonlinear mapping capability to model monthly HIV/AIDS incidence data in China from January 2001 to September 2025. Data were partitioned into training and validation sets at a 7:3 ratio, with data from January 2001 to June 2018 serving as the training set and data from July 2018 to September 2025 as the validation set. Grid search was used to optimize the penalty parameter C, the insensitive loss function parameter ε, and the kernel parameter γ, with Mean Squared Error (MSE) as the fitness function, to determine the optimal parameter combination and construct the SVR prediction model.

Model fitting results showed that the SVR model performed well on the validation set, with R² = 0.561, MAE = 0.497, and MAPE = 15.750%. SVR ranked second in R² (0.5610), only behind the LSTM-CNN parallel fusion model. These results indicate that the SVR model effectively captured the temporal variation trends of HIV/AIDS incidence in China and maintained high prediction stability under limited sample conditions.

**S3. Random Forest (RF) Model: Construction and Results**

**S3.1 RF Model Construction**

Random Forest is based on the Bootstrap Aggregating (Bagging) ensemble strategy, which constructs multiple independent decision trees and aggregates their results to mitigate overfitting risk (3). The model generates subsamples via Bootstrap sampling, constructs decision trees with random feature selection, and outputs the average of all decision tree predictions as the final result. Node splitting is based on the Gini index to select the optimal feature.

The Random Forest model was implemented on the MATLAB R2023b platform using the TreeBagger function from the Statistics and Machine Learning Toolbox. The number of trees (NumTrees) was determined via grid search over [100–500]; the maximum depth (max_depth) was searched over [5–30]; the minimum number of samples for split (min_samples_split) was searched over [2–10]; the minimum number of samples per leaf node (min_samples_leaf) was searched over [1–5]; and the maximum number of features (max_features) was searched over [sqrt, log2, None]. The optimal parameter combination was determined via two-stage grid search.

**Gini index:**

$\text{Gini}(D)=1-\sum_{j=1}^{J} p_{j}^{2}$	(4)

**Ensemble prediction:**

$\hat{y}=\frac{1}{B}\sum_{b=1}^{B} T_{b}(X)$	(5)

In formula (4-5): $p_{j}$ is the probability of a sample belonging to class$j$, and $J$ is the number of classes; $T_{b}(X)$ is the prediction result of the$b$ decision tree, and $B$ is the number of decision trees.

**S3.2 RF Model Results**

Random Forest is an efficient and versatile machine learning algorithm that combines the predictions of multiple decision trees using bagging, with each tree trained on a bootstrap subset of the data. Random feature selection at each node adds a layer of randomness, enhancing robustness against overfitting. The algorithm typically produces good results with minimal parameter tuning. Data were partitioned into training and validation sets at a 7:3 ratio, with data from January 2001 to June 2018 serving as the training set and data from July 2018 to September 2025 as the validation set.

Validation results showed that the Random Forest model exhibited poor fitting performance on the validation set. Its R² was 0.1983, MAE was 0.6782, and MAPE was 19.8900%.

**S4. Gated Recurrent Unit (GRU) Model: Construction and Results**

**S4.1 GRU Model Construction**

The Gated Recurrent Unit (GRU) captures temporal dependencies through update and reset gates, effectively alleviating the vanishing gradient problem (4). The update gate controls the extent to which the previous hidden state is retained, and the reset gate determines the degree to which previous information is forgotten.

The GRU model was implemented on the MATLAB R2023b platform using the Deep Learning Toolbox. The model adopted a two-layer GRU structure, and the number of hidden units was determined via grid search over [32, 64, 128]. A Dropout layer (with ratio determined via grid search over [0.1–0.5]) was incorporated to prevent overfitting. The final prediction was output via a fully connected layer. The Adam optimizer was used, with the initial learning rate determined via grid search over [0.0001–0.01]; batch size was searched over [16, 32, 64]; and training epochs were searched over [100–500].

**Update gate:**

$z_{t}=\sigma(W_{z}\cdot[h_{t-1},x_{t}]+b_{z})$	(6)

**Reset gate:**

$r_{t}=\sigma(W_{r}\cdot[h_{t-1},x_{t}]+b_{r})$	(7)

**Candidate hidden state:**

$\tilde{h}_{t}=tanh(W_{h}\cdot[r_{t}\odot h_{t-1},x_{t}]+b_{h})$	(8)

**Hidden state:**

$h_{t}=(1-z_{t})\odot h_{t-1}+z_{t}\odot\tilde{h}_{t}$	(9)

In formula (6-9): $z_{t}$ is the update gate, $r_{t}$ is the reset gate, $\tilde{h}_{t}$ is the candidate hidden state, and $h_{t}$ is the hidden state; $\sigma$ is the Sigmoid activation function,$\tanh$ is the hyperbolic tangent function, and$\odot$ denotes element-wise multiplication.

**S4.2 GRU Model Results**

GRU (Gated Recurrent Unit) is an improved variant of Recurrent Neural Network (RNN) that introduces update and reset gate mechanisms, effectively addressing the vanishing gradient problem faced by traditional RNNs in processing long sequences, while offering a more compact network structure compared to LSTM. The GRU model demonstrated certain fitting capability on the validation set, with R² = 0.493, MAE = 0.555, and MAPE = 16.950%. The relatively low MAPE value indicates good prediction stability in terms of percentage error; however, the R² value of only 0.493 suggests substantial room for improvement in the model's explanatory power for data variability.

**S5. Convolutional Neural Network (CNN) Model: Construction and Results**

**S5.1 CNN Model Construction**

Convolutional Neural Networks (CNN) extract local features and patterns from time series data through local connectivity and weight sharing (5). Convolutional operations achieve feature extraction by sliding convolution kernels over input data, while pooling operations reduce feature dimensionality and retain principal information.

The CNN model was implemented on the MATLAB R2023b platform using the Deep Learning Toolbox. The model was configured with three 1D convolutional layers; the number of convolution kernels was determined via grid search over [16, 32, 64, 128], and the kernel size was searched over [3, 5, 7, 12] (with kernel size = 12 specifically designed to directly capture annual seasonal cycles). The ReLU activation function was used, and each convolutional layer was followed by a MaxPooling1D layer (pooling size determined via grid search over [2, 3]). A Dropout layer (dropout rate determined via grid search over [0.1–0.5]) was added before the fully connected layer. The feature maps were then flattened via a Flatten layer and connected to two fully connected layers (number of neurons determined via grid search). The output layer used a linear activation function. The Adam optimizer was used (initial learning rate determined via grid search over [0.0001–0.01]), with Mean Squared Error (MSE) as the loss function; batch size was searched over [16, 32, 64]; and epochs were searched over [100–500].

**1D convolutional operation:**

$(f*g)(t)=\sum_{\tau=1}^{k} f(\tau)g(t-\tau)$	(10)

**Max pooling operation:**

$y_{i,j}=\max_{(m,n)\in\mathcal{R}_{i,j}} x_{m,n}$	(11)

In formula (10-11) : $k$ is the convolution kernel size, and $\mathcal{R}_{i,j}$ is the pooling window.

**S5.2 CNN Model Results**

CNN (Convolutional Neural Network) is a deep learning model whose core mechanism lies in automatically extracting temporal features and local patterns through the local perception properties of convolution kernels, combined with pooling operations to reduce feature dimensionality, effectively capturing nonlinear relationships and long-term dependency trends in time series data. Training was performed using data from January 2001 to June 2018 (210 monthly data points), and validation was conducted using data from July 2018 to September 2025 (87 monthly data points) to assess model generalization performance.

Specific validation set performance of the CNN model was as follows: R² = 0.3803, indicating that the model explained approximately 38.03% of data variability; MAE = 0.5713; and MAPE = 19.4900%. This MAPE value was within an acceptable range. CNN (R² = 0.3803) achieved moderate performance; its convolution kernel local perception properties effectively extracted monthly fluctuation patterns, but the lack of explicit temporal memory mechanisms made it difficult to capture long-term growth trends spanning multiple years.

**S6. Long Short-Term Memory (LSTM) Network: Construction and Results**

**S6.1 LSTM Model Construction**

Long Short-Term Memory (LSTM) networks address the vanishing gradient problem of traditional Recurrent Neural Networks through the design of forget gates, input gates, output gates, and cell states (6). The forget gate determines the information retained in the cell state, the input gate controls the input of new information, and the output gate regulates the output of the hidden state.

The LSTM model was implemented on the MATLAB R2023b platform using the Deep Learning Toolbox. The model adopted a two-layer LSTM structure, with the first layer containing 64 neurons and returning sequence outputs, and the second layer containing 32 neurons. To accelerate network convergence and prevent overfitting, a Batch Normalization layer (whether to use was determined via grid search over [True, False]) and a Dropout layer (dropout rate determined via grid search over [0.1–0.5]) were added. The output layer was a fully connected layer using a linear activation function for prediction. The Adam optimizer was used (initial learning rate determined via grid search over [0.0001–0.01]), with Mean Squared Error (MSE) as the loss function; training epochs were searched over [100–500]; batch size was searched over [16, 32, 64]; and early stopping (patience determined via grid search over [10, 20]) was introduced. The time step (lookback) was determined via grid search over [3, 6, 12, 24].

**Forget gate:**

$f_{t}=\sigma(W_{f}\cdot[h_{t-1},x_{t}]+b_{f})$	(12)

**Input gate:**

$i_{t}=\sigma(W_{i}\cdot[h_{t-1},x_{t}]+b_{i})$	(13)

**Candidate cell state:**

$\tilde{C}_{t}=tanh(W_{C}\cdot[h_{t-1},x_{t}]+b_{C})$	(14)

**Cell state:**

$C_{t}=f_{t}\odot C_{t-1}+i_{t}\odot\tilde{C}_{t}$	(15)

**Hidden state:**

$h_{t}=o_{t}\odot tanh(C_{t})$	(16)

In formula (12-16) : $f_{t}$ is the forget gate, $i_{t}$ is the input gate, $o_{t}$ is the output gate, $C_{t}$ is the cell state, $h_{t}$ is the hidden state, and $\tilde{C}_{t}$ is the candidate cell state;$\sigma$ is the Sigmoid activation function, and $\odot$ denotes element-wise multiplication.

**S6.2 LSTM Model Results**

LSTM (Long Short-Term Memory) is an improved Recurrent Neural Network that introduces forget gates, input gates, and output gate mechanisms, effectively addressing the vanishing gradient problem of traditional RNNs and thereby capturing long-term dependencies in time series data. It is suitable for prediction tasks with complex nonlinear dynamic characteristics.

Training was performed using monthly HIV/AIDS incidence data in China from January 2001 to June 2018. The trained LSTM model was used to predict validation set data from July 2018 to September 2025. The model demonstrated certain fitting capability, with validation set R² = 0.335, MAE = 0.599, and MAPE = 17.930%.

**S7. GRU-LSTM Serial Fusion Model: Construction and Results**

**S7.1 GRU-LSTM Serial Fusion Model Construction**

The GRU-LSTM serial fusion model combines GRU and LSTM in a serial architecture, in which GRU first extracts shallow temporal features and LSTM subsequently captures deep long-term dependencies, achieving hierarchical feature learning (7). The GRU output serves as the LSTM input, and the final deep hidden state at the last time step is passed through a fully connected layer to produce the prediction.

The GRU-LSTM serial fusion model was implemented on the MATLAB R2023b platform using the Deep Learning Toolbox. The model structure adopted a time step (lookback) determined via grid search over [3, 6, 12, 24]. First, a GRU layer (number of units determined via grid search over [32, 64, 128]) extracted short-term dynamic features; subsequently, the GRU output was fed into an LSTM layer (number of units determined via grid search over [32, 64, 128]). The Adam optimizer was used (learning rate determined via grid search over [0.0001–0.01]), with Mean Squared Error (MSE) as the loss function; batch size was searched over [16, 32, 64]; epochs were searched over [100–500]; and early stopping (patience determined via grid search over [10, 20]) was introduced.

**GRU output as LSTM input:**

$h_{t}^{(G)}=\text{GRU}(x_{t},h_{t-1}^{(G)})$	(17)

**LSTM final hidden state:**

$\hat{y}=W_{o}\cdot h_{T}^{(L)}+b_{o}$	(18)

In formula (17-18) : $h_{t}^{(G)}$ is the hidden state of GRU at time t; $h_{T}^{(L)}$ is the final hidden state of LSTM, $W_{o}$ is the output weight matrix, and $b_{o}$ is the bias term.

**S7.2 GRU-LSTM Serial Fusion Model Results**

GRU-LSTM (Gated Recurrent Unit–Long Short-Term Memory) is a hybrid deep learning model that effectively captures long-term dependencies and complex nonlinear features in time series data by combining the update gate mechanism of GRU with the memory cell structure of LSTM. Training was performed using monthly HIV/AIDS incidence data in China from January 2001 to June 2018 (210 monthly observations), and validation was conducted using data from July 2018 to September 2025 (87 monthly observations) to ensure sufficient sample size for parameter learning and performance validation.

Through comprehensive analysis and comparison, the GRU-LSTM model demonstrated stable predictive performance on the validation set. Its R² was 0.479, MAE was 0.554, and MAPE was 17.400%. The GRU-LSTM serial fusion (R² = 0.479) outperformed the single LSTM (0.335), although the improvement was limited. The advantage of serial fusion lies in hierarchical feature extraction: the compact structure of GRU reduces computational overhead, while the fine-grained gating of LSTM enhances long-term memory capacity. These metrics indicate that the GRU-LSTM model can capture the temporal variation patterns of monthly HIV/AIDS incidence data in China to a certain extent, exhibiting certain fitting and predictive capabilities for public health time series data.

**S8. LSTM-CNN Parallel Fusion Model: Construction and Results**

**S8.1 LSTM-CNN Parallel Fusion Model Construction**

The LSTM-CNN parallel fusion model combines LSTM and CNN in a parallel architecture, in which the CNN branch extracts local temporal features while the LSTM branch captures long-term temporal dependencies; the fused dual-branch features enable precise prediction (8). The dual-branch features are concatenated and passed to fully connected layers.

The LSTM-CNN parallel fusion model was implemented on the MATLAB R2023b platform using the Deep Learning Toolbox. The model contains two parallel pathways: (1) CNN pathway: a Conv1D layer (number of filters determined via grid search over [16, 32, 64], kernel size determined via grid search over [3, 5, 7, 12], with kernel size = 12 specifically designed to directly capture annual seasonal cycles) and MaxPooling layer (pooling size determined via grid search over [2, 3]) for feature extraction and dimensionality reduction; (2) LSTM pathway: an LSTM layer (number of units determined via grid search over [32, 64, 128]) to capture temporal dependencies. The dual-branch features were fused via concatenation or addition (fusion method determined via grid search over [concatenate, add]), followed by a fusion layer (number of units determined via grid search over [64, 128, 256]) for further processing. A Dropout layer (dropout rate determined via grid search over [0.1–0.5]) was added to prevent overfitting, and the final prediction was output by a Dense layer using a linear activation function to ensure unrestricted regression output range. The ReLU activation function was used for hidden layers. The Adam optimizer was used (learning rate determined via grid search over [0.0001–0.01]). Batch size was searched over [16, 32, 64]; epochs were searched over [100–500]; and early stopping (patience determined via grid search over [10, 20]) was introduced. The time step (lookback) was determined via grid search over [12, 24].

**CNN local features:**

$F_{CNN}=\text{CNN}(X)$	(19)

**LSTM temporal features:**

$F_{LSTM}=\text{LSTM}(X)$	(20)

**Fused features:**

$F_{fuse}=\text{Concat}(F_{CNN},F_{LSTM})$	(21)

**Prediction output:**

$\hat{y}=W_{f}\cdot F_{fuse}+b_{f}$	(22)

In formula (19-22) : $F_{CNN}$ represents the local features extracted by CNN, $F_{LSTM}$ represents the temporal features extracted by LSTM ,$F_{fuse}$ represents the fused features, $W_{f}$ is the fusion weight matrix, and $b_{f}$ is the bias term.

**S8.2 LSTM-CNN Parallel Fusion Model Results**

This study constructed the LSTM-CNN parallel fusion model using the MATLAB R2023b platform and the Deep Learning Toolbox. The model contains two parallel pathways: the CNN pathway extracts local features from the input data, and the LSTM pathway learns long-term temporal dependencies. The outputs of both pathways are merged via a concatenation layer and passed through fully connected layers for final prediction. Training was performed using monthly HIV/AIDS incidence data in China from January 2001 to June 2018, with appropriate lookback steps to construct input sequences. Data were simultaneously fed into the CNN and LSTM pathways via batch processing. After sufficient training, predictions were made on the validation set data from July 2018 to September 2025.

Through comprehensive analysis and comparison, we found that the LSTM-CNN parallel fusion model excelled across all evaluation metrics. Its R² reached 0.605, MAE was only 0.456, and MAPE was as low as 14.690%. These metrics were optimal among all fitted models, with the highest R² and the lowest MAE and MAPE. Compared with single LSTM, single CNN, SARIMA, SVR, and other models, the LSTM-CNN parallel fusion model effectively leveraged the advantages of CNN in local feature extraction and LSTM in long-term dependency capture through its parallel architecture, substantially enhancing prediction accuracy.

**S9. LSTM-GRU-Attention Fusion Model: Construction and Results**

**S9.1 LSTM-GRU-Attention Fusion Model Construction**

The LSTM-GRU-Attention fusion model integrates LSTM, GRU, and temporal attention mechanisms. The hidden states from the LSTM and GRU dual branches are concatenated as temporal features, and the attention mechanism subsequently strengthens the feature weights of key time steps (9). Monthly HIV/AIDS incidence data in China from January 2001 to September 2025 were used, with data from January 2001 to June 2018 for model training and data from July 2018 to September 2025 for validation.

The LSTM-GRU-Attention fusion model was implemented on the MATLAB R2023b platform using the Deep Learning Toolbox. The Adam optimizer (learning rate determined via grid search over [0.0001–0.01]) and Mean Squared Error (MSE) loss function were used for training (the original manuscript used MAE, which has now been unified to MSE to ensure fair comparison across models). Early stopping (patience determined via grid search over [10, 20]) was introduced to prevent overfitting. The network architecture included an LSTM layer (number of units determined via grid search over [32, 64, 128]), a GRU layer (number of units determined via grid search over [32, 64, 128]), and a custom attention layer (number of attention heads determined via grid search over [1, 2, 4], attention dimension determined via grid search over [16, 32, 64]), extracting temporal features in a hierarchical progressive manner. Batch size was searched over [16, 32, 64]; epochs were searched over [100–500]; and the time step (lookback) was determined via grid search over [6, 12, 24].

**Attention weight calculation:**

$e_{t}=v^{T}tanh(W_{h}h_{t}+b_{a}), \alpha_{t}=\frac{exp(e_{t})}{\sum_{j=1}^{T} exp(e_{j})}$	(23)

**Context vector:**

$c=\sum_{t=1}^{T} \alpha_{t}h_{t}$	(24)

**Prediction output:**

$\hat{y}=W_{o}\cdot c+b_{o}$	(25)

In formula (23-25) : $\alpha_{t}$ is the attention weight, $c$ is the context vector, $v$、$W_{h}$ and $W_{o}$ are weight matrices, and $b_{a}$ and $b_{o}$ are bias terms.

**S9.2 LSTM-GRU-Attention Fusion Model Results**

LSTM (Long Short-Term Memory) and GRU (Gated Recurrent Unit) are improved Recurrent Neural Network (RNN) architectures that effectively learn long-term dependencies in time series data through gating mechanisms. The Attention mechanism dynamically allocates weights, enabling the model to focus on historically critical time steps for the current prediction. This study combined LSTM, GRU, and the Attention mechanism using monthly HIV/AIDS incidence data in China from January 2001 to September 2025, with data from January 2001 to June 2018 for training and data from July 2018 to September 2025 for validation. Validation set evaluation results showed that this hybrid model exhibited certain fitting performance. Its R² was 0.335, MAE was 0.568, and MAPE was 18.070%.

**S10. LSTM-CNN-Attention Fusion Model: Construction and Results**

**S10.1 LSTM-CNN-Attention Fusion Model Construction**

The LSTM-CNN-Attention model is a fused deep learning approach that extracts temporal features from time series data using Long Short-Term Memory (LSTM), mines local spatial features using Convolutional Neural Network (CNN), and introduces the Attention mechanism to enhance focus on critical time steps, thereby achieving high-precision prediction of complex time series data .

The LSTM-CNN-Attention fusion model was implemented on the MATLAB R2023b platform using the Deep Learning Toolbox. A parallel architecture was adopted to simultaneously extract temporal and spatial features, with the Attention mechanism performing weighted fusion of multi-time-step features. The LSTM layer included units determined via grid search over [32, 64, 128]; the CNN layer adopted a number of convolutional layers determined via grid search over [1, 2, 3], with the number of filters per layer determined via grid search over [16, 32, 64] and kernel size determined via grid search over [3, 5, 7, 12]; the attention mechanism adopted a number of attention heads determined via grid search over [1, 2, 4]. The Adam optimizer was used (initial learning rate determined via grid search over [0.0001–0.01]), with Mean Squared Error (MSE) as the loss function; early stopping patience was determined via grid search over [10, 20]; maximum training epochs were searched over [100–500].

**Spatiotemporal attention weights:**

$e_{t,f}=v^{T}tanh(W_{f}[F_{CNN};F_{LSTM}]+b_{s}), \alpha_{t,f}=\frac{exp(e_{t,f})}{\sum_{i,j} exp(e_{i,j})}$	(26)

**Weighted features:**

$F_{\mathrm{attn}}=\sum_{t} \sum_{f} \alpha_{t,f}\cdot[F_{\mathrm{CNN}};F_{\mathrm{LSTM}}]_{t,f}$	(27)

**Prediction output:**

$\hat{y}=W_{o}\cdot F_{attn}+b_{o}$	(28)

In formula (26-28) : $\alpha_{t,f}$ is the spatiotemporal attention weight, $F_{\mathrm{attn}}$ is the weighted feature, $[F_{\mathrm{CNN}};F_{\mathrm{LSTM}}]$ is the concatenated feature, and $W_{o}$ and $b_{o}$ are output layer parameters.

**S10.2 LSTM-CNN-Attention Fusion Model Results**

The LSTM-CNN-Attention model is a fused deep learning approach that extracts temporal features from time series data using Long Short-Term Memory (LSTM), mines local spatial features using Convolutional Neural Network (CNN), and introduces the Attention mechanism to enhance focus on critical time steps, thereby achieving high-precision prediction of complex time series data. Training was performed using monthly HIV/AIDS incidence data in China from January 2001 to June 2018 to construct the LSTM-CNN-Attention fusion prediction model. Data from July 2018 to September 2025 were used for validation.

According to comprehensive analysis, the LSTM-CNN-Attention fusion model achieved R² = 0.477, MAE = 0.528, and MAPE = 15.640% on the validation set. LSTM-CNN-Attention (R² = 0.4768) exhibited a gap compared with LSTM-CNN parallel (0.6053), indicating that under the conditions of this study, the incremental benefit of the attention mechanism was limited.(3)

**References**

1. Yang L, Zhang W, Wang C, Ma J, Huang Y, Yao Y, Wang K. Multiple change point analysis and time series forecast of brucellosis reports in Xinjiang, China from 2010 to 2024. BMC Public Health. 2025;25(1):3365.DOI:10.1186/s12889-025-24473-7

2.Liu J, Shi G, Zhu K. Vessel trajectory prediction model based on AIS sensor data and adaptive chaos differential evolution support vector regression (ACDE-SVR). Appl Sci. (2019) 9:2983. doi: 10.3390/app9142983

3. Adewole KS, Jacobsson A, Davidsson P. Intrusion Detection Framework for Internet of Things with Rule Induction for Model Explanation. Sensors (Basel). 2025;25(6).DOI:10.3390/s25061845

4.Wu H, Dong H, He Y, Duan Q. Smart contract vulnerability detection based on hybrid attention mechanism model. Appl Sci. (2023) 13:770. doi: 10.3390/app13020770

5.Rani S, Rout A, Soni P, Gupta M, Kumar N, Kumar K. Review of CNN-based approaches for preprocessing, segmentation and classification of knee osteoarthritis. Diagnostics. (2026) 16:461. doi: 10.3390/diagnostics16030461

6.Chen WT, Wu HL, Ren SY. CM-LSTM based spectrum sensing. Sensors (2022) 22:2286. doi:10.3390/s22062286

7. Abade A, Porto LF, Scholze AR, Kuntath D, Barros NDS, Berra TZ, et al. A comparative analysis of classical and machine learning methods for forecasting TB/HIV co-infection. Sci Rep. 2024;14(1):18991.DOI:10.1038/s41598-024-69580-4

8.Farsi B, Amayri M, Bouguila N, Eicker U. On short-term load forecasting using machine learning techniques and a novel parallel deep LSTM-CNN approach. IEEE Access (2021) 9:31191-31212. doi:10.1109/ACCESS.2021.3060290

9.Mustafa G, Hwang Y, Cho SJ. Predicting bus travel time in Cheonan City through deep learning utilizing digital tachograph data. Electronics (2024) 13(9):1771. doi:10.3390/electronics13091771
