# Supplementary material for: Forecasting monthly AIDS incidence in China via LSTM-CNN parallel fusion: a comparative study of 10 predictive models
Source: Front Public Health. 2026 Jul 29;14:1873637. doi: 10.3389/fpubh.2026.1873637 (PMC13461732; doi:10.3389/fpubh.2026.1873637)
Supplement: Supplementary file 2 [file Supplementary_file_2.docx]

**Supplementary Material（attachment 2）**

**Forecasting Monthly AIDS Incidence in China via LSTM-CNN Parallel Fusion: A Comparative Study of Ten Predictive Models**

Chengcheng Li^1†^ , Jinfeng Li^2†^ , Shifeng Pang^3†^ , Chao Rong^1^, Ximan Ye^2^, Xiaojie Lao^4*^, Maowei Chen^2*^

^1^ Humanities and Management School, Zhejiang Chinese Medical University, Hangzhou, Zhejiang, China,

^2^ Department of Infectious Diseases, Wuming Hospital Affiliated to Guangxi Medical University, Nanning, Guangxi, China

^3^ Department of Cardiology, Minzu Hospital of Guangxi Zhuang Autonomous Region, Nanning, Guangxi, China

^4^ Department of Cardiology, Sir Run Run Shaw Hospital, Zhejiang University School of Medicine, Hangzhou, Zhejiang, China

**^†^** Chengcheng Li, Jinfeng Li, and Shifeng Pang contributed equally to this study

*** Correspondence:**

Xiaojie Lao

No. 3, Qingchun East Road, Shangcheng District, Hangzhou, Zhejiang Province

Email: xiaojielao@126.com (X. Lao), ORCID iD: 0009-0009-4675-6480

Maowei Chen

No. 26 Yongning Road, Wuming District, Nanning, Guangxi, China

Email: chenmaowei2008@163.com (M. Chen), ORCID iD: 0000-0001-8673-4328

Total pages: 5 (including cover page)

**Predicting Monthly HIV/AIDS Incidence in China Using an LSTM-CNN (Parallel) Model: A Comparative Study of Ten Forecasting Algorithms**

Authors, Affiliations

Total pages: 4 (including cover page)

**Figure S2. LSTM-CNN parallel fusion model prediction of monthly AIDS incidence in China with 95% confidence intervals (November 2025–September 2035).** The solid blue line denotes the point estimate of predicted incidence (per million population), and the shaded blue region represents the 95% confidence interval. Selected annual peak values are annotated on the curve to illustrate the projected seasonal fluctuation pattern.

**Table S1. Ten-Year Ahead Forecast of Monthly AIDS Incidence in China Based on the LSTM-CNN Parallel Fusion Model**

**(November 2025 – September 2035)**

| **Forecast month** | **Predicted incidence (per million)** | **95% CI lower bound** | **95% CI upper bound** |
| --- | --- | --- | --- |
| Oct 2025 | 3.459 | 3.095 | 3.822 |
| Nov 2025 | 3.024 | 2.660 | 3.388 |
| Dec 2025 | 2.362 | 1.998 | 2.726 |
| Jan 2026 | 2.865 | 2.501 | 3.228 |
| Feb 2026 | 3.319 | 2.955 | 3.683 |
| Mar 2026 | 3.143 | 2.779 | 3.507 |
| Apr 2026 | 3.062 | 2.698 | 3.426 |
| May 2026 | 3.359 | 2.996 | 3.723 |
| Jun 2026 | 3.217 | 2.853 | 3.581 |
| Jul 2026 | 3.104 | 2.740 | 3.468 |
| Aug 2026 | 3.150 | 2.786 | 3.514 |
| Sep 2026 | 3.369 | 3.006 | 3.733 |
| Oct 2026 | 3.435 | 3.071 | 3.798 |
| Nov 2026 | 2.967 | 2.603 | 3.331 |
| Dec 2026 | 2.617 | 2.253 | 2.981 |
| Jan 2027 | 3.152 | 2.788 | 3.515 |
| Feb 2027 | 3.325 | 2.961 | 3.688 |
| Mar 2027 | 3.178 | 2.814 | 3.542 |
| Apr 2027 | 3.061 | 2.698 | 3.425 |
| May 2027 | 3.203 | 2.839 | 3.566 |
| Jun 2027 | 3.230 | 2.866 | 3.594 |
| Jul 2027 | 3.269 | 2.905 | 3.632 |
| Aug 2027 | 3.302 | 2.938 | 3.666 |
| Sep 2027 | 3.423 | 3.059 | 3.787 |
| Oct 2027 | 3.341 | 2.977 | 3.705 |
| Nov 2027 | 3.042 | 2.678 | 3.406 |
| Dec 2027 | 2.997 | 2.633 | 3.361 |
| Jan 2028 | 3.345 | 2.981 | 3.709 |
| Feb 2028 | 3.364 | 3.000 | 3.728 |
| Mar 2028 | 3.169 | 2.806 | 3.533 |
| Apr 2028 | 3.059 | 2.696 | 3.423 |
| May 2028 | 3.220 | 2.856 | 3.584 |
| Jun 2028 | 3.333 | 2.969 | 3.697 |
| Jul 2028 | 3.363 | 2.999 | 3.727 |
| Aug 2028 | 3.327 | 2.963 | 3.691 |
| Sep 2028 | 3.374 | 3.010 | 3.738 |
| Oct 2028 | 3.313 | 2.949 | 3.676 |
| Nov 2028 | 3.223 | 2.859 | 3.587 |
| Dec 2028 | 3.280 | 2.916 | 3.644 |
| Jan 2029 | 3.425 | 3.061 | 3.789 |
| Feb 2029 | 3.350 | 2.986 | 3.714 |
| Mar 2029 | 3.195 | 2.831 | 3.558 |
| Apr 2029 | 3.185 | 2.821 | 3.549 |
| May 2029 | 3.337 | 2.973 | 3.700 |
| Jun 2029 | 3.413 | 3.049 | 3.777 |
| Jul 2029 | 3.382 | 3.019 | 3.746 |
| Aug 2029 | 3.320 | 2.956 | 3.684 |
| Sep 2029 | 3.355 | 2.991 | 3.718 |
| Oct 2029 | 3.368 | 3.004 | 3.732 |
| Nov 2029 | 3.372 | 3.008 | 3.735 |
| Dec 2029 | 3.401 | 3.037 | 3.765 |
| Jan 2030 | 3.424 | 3.060 | 3.787 |
| Feb 2030 | 3.339 | 2.975 | 3.703 |
| Mar 2030 | 3.284 | 2.920 | 3.648 |
| Apr 2030 | 3.330 | 2.966 | 3.694 |
| May 2030 | 3.421 | 3.058 | 3.785 |
| Jun 2030 | 3.433 | 3.069 | 3.796 |
| Jul 2030 | 3.367 | 3.003 | 3.730 |
| Aug 2030 | 3.337 | 2.973 | 3.701 |
| Sep 2030 | 3.401 | 3.037 | 3.765 |
| Oct 2030 | 3.441 | 3.077 | 3.805 |
| Nov 2030 | 3.443 | 3.079 | 3.807 |
| Dec 2030 | 3.429 | 3.065 | 3.793 |
| Jan 2031 | 3.412 | 3.048 | 3.775 |
| Feb 2031 | 3.381 | 3.018 | 3.745 |
| Mar 2031 | 3.393 | 3.029 | 3.757 |
| Apr 2031 | 3.431 | 3.067 | 3.795 |
| May 2031 | 3.456 | 3.092 | 3.819 |
| Jun 2031 | 3.422 | 3.059 | 3.786 |
| Jul 2031 | 3.382 | 3.018 | 3.745 |
| Aug 2031 | 3.402 | 3.038 | 3.765 |
| Sep 2031 | 3.463 | 3.099 | 3.827 |
| Oct 2031 | 3.480 | 3.117 | 3.844 |
| Nov 2031 | 3.458 | 3.094 | 3.822 |
| Dec 2031 | 3.434 | 3.070 | 3.798 |
| Jan 2032 | 3.434 | 3.070 | 3.798 |
| Feb 2032 | 3.451 | 3.087 | 3.815 |
| Mar 2032 | 3.470 | 3.106 | 3.834 |
| Apr 2032 | 3.473 | 3.109 | 3.837 |
| May 2032 | 3.460 | 3.096 | 3.824 |
| Jun 2032 | 3.433 | 3.070 | 3.797 |
| Jul 2032 | 3.436 | 3.072 | 3.800 |
| Aug 2032 | 3.472 | 3.109 | 3.836 |
| Sep 2032 | 3.500 | 3.136 | 3.864 |
| Oct 2032 | 3.487 | 3.124 | 3.851 |
| Nov 2032 | 3.461 | 3.098 | 3.825 |
| Dec 2032 | 3.458 | 3.094 | 3.822 |
| Jan 2033 | 3.485 | 3.121 | 3.849 |
| Feb 2033 | 3.505 | 3.141 | 3.869 |
| Mar 2033 | 3.502 | 3.138 | 3.865 |
| Apr 2033 | 3.485 | 3.121 | 3.848 |
| May 2033 | 3.472 | 3.108 | 3.836 |
| Jun 2033 | 3.475 | 3.112 | 3.839 |
| Jul 2033 | 3.497 | 3.134 | 3.861 |
| Aug 2033 | 3.513 | 3.150 | 3.877 |
| Sep 2033 | 3.510 | 3.146 | 3.874 |
| Oct 2033 | 3.490 | 3.127 | 3.854 |
| Nov 2033 | 3.485 | 3.121 | 3.848 |
| Dec 2033 | 3.505 | 3.141 | 3.869 |
| Jan 2034 | 3.528 | 3.164 | 3.892 |
| Feb 2034 | 3.528 | 3.164 | 3.891 |
| Mar 2034 | 3.511 | 3.147 | 3.875 |
| Apr 2034 | 3.499 | 3.135 | 3.863 |
| May 2034 | 3.507 | 3.143 | 3.871 |
| Jun 2034 | 3.525 | 3.161 | 3.889 |
| Jul 2034 | 3.534 | 3.170 | 3.898 |
| Aug 2034 | 3.528 | 3.164 | 3.892 |
| Sep 2034 | 3.516 | 3.152 | 3.880 |
| Oct 2034 | 3.512 | 3.148 | 3.876 |
| Nov 2034 | 3.527 | 3.163 | 3.890 |
| Dec 2034 | 3.544 | 3.180 | 3.908 |
| Jan 2035 | 3.547 | 3.183 | 3.911 |
| Feb 2035 | 3.534 | 3.171 | 3.898 |
| Mar 2035 | 3.524 | 3.160 | 3.888 |
| Apr 2035 | 3.531 | 3.167 | 3.894 |
| May 2035 | 3.548 | 3.184 | 3.911 |
| Jun 2035 | 3.555 | 3.191 | 3.919 |
| Jul 2035 | 3.548 | 3.184 | 3.912 |
| Aug 2035 | 3.537 | 3.173 | 3.901 |
| Sep 2035 | 3.536 | 3.172 | 3.900 |

Abbreviation: CI = confidence interval.
