# Supplementary material for: Forecasting monthly AIDS incidence in China via LSTM-CNN parallel fusion: a comparative study of 10 predictive models
Source: Front Public Health. 2026 Jul 29;14:1873637. doi: 10.3389/fpubh.2026.1873637 (PMC13461732; doi:10.3389/fpubh.2026.1873637)
Supplement: Supplementary file 3 [file Supplementary_file_3.docx]

**Supplementary Material（attachment 3）**

**Forecasting Monthly AIDS Incidence in China via LSTM-CNN Parallel Fusion: A Comparative Study of Ten Predictive Models**

Chengcheng Li^1†^ , Jinfeng Li^2†^ , Shifeng Pang^3†^ , Chao Rong^1^, Ximan Ye^2^, Xiaojie Lao^4*^, Maowei Chen^2*^

^1^ Humanities and Management School, Zhejiang Chinese Medical University, Hangzhou, Zhejiang, China,

^2^ Department of Infectious Diseases, Wuming Hospital Affiliated to Guangxi Medical University, Nanning, Guangxi, China

^3^ Department of Cardiology, Minzu Hospital of Guangxi Zhuang Autonomous Region, Nanning, Guangxi, China

^4^ Department of Cardiology, Sir Run Run Shaw Hospital, Zhejiang University School of Medicine, Hangzhou, Zhejiang, China

**^†^** Chengcheng Li, Jinfeng Li, and Shifeng Pang contributed equally to this study

*** Correspondence:**

Xiaojie Lao

No. 3, Qingchun East Road, Shangcheng District, Hangzhou, Zhejiang Province

Email: xiaojielao@126.com (X. Lao), ORCID iD: 0009-0009-4675-6480

Maowei Chen

No. 26 Yongning Road, Wuming District, Nanning, Guangxi, China

Email: chenmaowei2008@163.com (M. Chen), ORCID iD: 0000-0001-8673-4328

Total pages: 6 (including cover page)

**S11：**Final Architecture and Selected Hyperparameters of the LSTM-CNN Parallel Fusion Model

The LSTM-CNN parallel fusion model was constructed using MATLAB R2023b (Deep Learning Toolbox) with a layerGraph architecture. The input layer accepts a time-window of 12 consecutive monthly incidence values (lag = 12), formatted as a 2-D tensor (batch_size, 12, 1). The architecture comprises two parallel branches whose outputs are concatenated and fed into a fully connected output layer.

LSTM Branch (Temporal Dependency Modeling):
• LSTM Layer 1: 100 hidden units, OutputMode = "sequence", preserving temporal sequences for deeper feature extraction.
• Dropout Layer: rate = 0.2, for mitigating overfitting.
• LSTM Layer 2: 50 hidden units, OutputMode = "last", outputting the final hidden state.
• Fully Connected Layer: 64 units, projecting the LSTM output to the fusion dimension.

CNN Branch (Global Feature Extraction):
• Fully Connected Layer 1: 128 units.
• ReLU Activation Layer.
• Dropout Layer: rate = 0.2.
• Fully Connected Layer 2: 64 units (128/2).
• ReLU Activation Layer.
• Fully Connected Layer 3: 64 units, projecting to the fusion dimension.

Fusion and Output Head:
• Concatenation Layer: the 64-dimensional output from the LSTM branch and the 64-dimensional output from the CNN branch are concatenated along the feature axis, producing a 128-dimensional fused feature vector.
• Fully Connected Output Layer: 1 unit, outputting the forecasted normalized incidence value for the next month.
• Regression Output Layer: for continuous value prediction.

Training Configuration:
• Optimizer: Adam (adaptive moment estimation).
• Initial learning rate: 0.001.
• Loss function: Mean Squared Error (MSE).
• Batch size: determined automatically by MATLAB based on the training data size.
• Maximum epochs: 800.
• Early stopping: monitored on validation loss (MSE), patience = 30 epochs, restoring the best weights.
• Learning rate schedule: piecewise decay, with a drop factor of 0.5 applied every 75 epochs.
• Validation frequency: every 10 iterations.
• Shuffle: every epoch.
• Execution environment: auto (CPU/GPU).

Data Preprocessing:
• Z-score standardization: data were normalized to zero mean and unit variance prior to model training.
• Data augmentation: Gaussian noise (noise_factor = 0.01) was added to the training features, doubling the training sample size to improve model robustness.
• Train-validation split: 70% of the observations for training, 30% for validation (chronological split).

Ensemble Strategy:
• Repeated training: the model was independently trained 5 times with different random initializations.
• Final prediction: the arithmetic mean of the 5 independent model predictions was used as the final forecast to reduce variance and enhance stability.

Hyperparameter Selection:
The above hyperparameters were determined through systematic manual tuning guided by validation MSE. Key hyperparameters (LSTM hidden units, CNN layer dimensions, dropout rate, learning rate, and lag window) were iteratively adjusted within plausible ranges, and the configuration yielding the lowest validation MSE was selected as the final architecture.

**S12：Pseudocode: LSTM-CNN Parallel Fusion Model**

This pseudocode details the complete workflow of the LSTM-CNN parallel fusion model for time series forecasting, covering data preprocessing, model architecture definition, repeated training, evaluation, and future forecasting.

Algorithm 1: Parallel Fusion LSTM-CNN Model Training and Forecasting

Input:
D: Raw time series data, D = {d_1, d_2, ..., d_T}
w: Time step (look-back window)
ρ: Training set split ratio
N_repeat: Number of repeated training runs
H_forecast: Horizon for future forecasting

Output:
Y_forecast: Forecasted values for the next H_forecast steps
Metrics: Model evaluation metrics (R², MAE, MAPE) on training and validation sets

1. Data Preprocessing and Preparation
   μ, σ ← Mean(D), Std(D)
   D_scaled ← (D - μ) / σ // Z-score normalization

// Create supervised learning dataset
X, Y ← CreateSlidingWindow(D_scaled, w)
// Shape of X is (num_samples, w), Y is the corresponding target value for the next time step

// Split into training and validation sets
N_train ← Floor(Length(X) × ρ)
X_train, Y_train ← X[1 : N_train], Y[1 : N_train]
X_val, Y_val ← X[N_train+1 : end], Y[N_train+1 : end]

// Data augmentation: Add Gaussian noise to the training set
X_train_aug, Y_train_aug ← AugmentData(X_train, Y_train)

2. Define Parallel Fusion Network Architecture
   Define input layer Input with dimension w

// Branch 1: Stacked LSTM Branch
LSTM_1 ← LSTMLayer(units=100, return_sequences=True)
Dropout_1 ← DropoutLayer(rate=0.2)
LSTM_2 ← LSTMLayer(units=50, return_sequences=False)
CNN_LSTM ← CNNLayer(units=64)
Output_LSTM ← CNN_LSTM(Dropout_1(LSTM_2(Dropout_1(LSTM_1(Input)))))

// Branch 2: CNN Branch
CNN_1 ← CNNLayer(units=128)
ReLU_1 ← ReLULayer()
Dropout_2 ← DropoutLayer(rate=0.2)
CNN_2 ← CNNLayer(units=64)
ReLU_2 ← ReLULayer()
CNN_Final ← CNNLayer(units=64)
Output_CNN ← CNN_Final(ReLU_2(CNN_2(Dropout_2(ReLU_1(CNN_1(Input))))))

// Feature Fusion and Output
Concat ← Concatenate(Output_LSTM, Output_CNN)
Final_CNN ← CNNLayer(units=1)
Output ← Final_CNN(Concat)

Construct model M with Input and Output

3. Repeated Training and Model Ensembling
   Initialize prediction storage matrix P_val with dimensions (Length(X_val), N_repeat)

For i = 1 to N_repeat do
Initialize model M_i with the architecture defined above
Configure optimizer (Adam), learning rate schedule, and early stopping
M_i ← Train(M_i, X_train_aug, Y_train_aug, X_val, Y_val)
P_val[:, i] ← Predict(M_i, X_val)
End For

P_val_avg ← Mean(P_val, axis=1) // Average the predictions from N_repeat runs
M_final ← M_i // Save the last trained model for future forecasting

4. Model Evaluation
   // Inverse transform predictions and actual values
   Y_val_orig ← Y_val * σ + μ
   P_val_orig ← P_val_avg * σ + μ

Calculate validation metrics: R²_val, MAE_val, MAPE_val ← Evaluate(Y_val_orig, P_val_orig)

// Calculate training set metrics similarly
P_train_scaled ← Predict(M_final, X_train)
P_train_orig ← P_train_scaled * σ + μ
Y_train_orig ← Y_train * σ + μ
Calculate training metrics: R²_train, MAE_train, MAPE_train ← Evaluate(Y_train_orig, P_train_orig)

5. Future Forecasting
   Initialize Input_buffer ← D_scaled[end-w+1 : end]
   Initialize Y_forecast_scaled as an empty list

For t = 1 to H_forecast do
y_pred_scaled ← Predict(M_final, Input_buffer)
Append y_pred_scaled to Y_forecast_scaled
Input_buffer ← UpdateBuffer(Input_buffer, y_pred_scaled) // Slide the window
End For

Y_forecast ← Y_forecast_scaled * σ + μ // Inverse transform to get final forecast

Return Y_forecast, Metrics

**Figure S3. Architecture of the CNN-LSTM parallel fusion model for monthly AIDS incidence prediction.** The CNN-LSTM parallel fusion model first extracts local features from monthly AIDS time-series data, and then employs LSTM to learn temporal dependencies to realize time-series forecasting of infectious disease incidence.
